# Ubiquitination-coupled liquid phase separation regulates the accumulation of the TRIM family of ubiquitin ligases into cytoplasmic bodies

**DOI:** 10.1371/journal.pone.0272700

**Published:** 2022-08-05

**Authors:** Takafumi Tozawa, Kohichi Matsunaga, Tetsuro Izumi, Naotake Shigehisa, Takamasa Uekita, Masato Taoka, Tohru Ichimura

**Affiliations:** 1 Department of Applied Chemistry, National Defense Academy, Yokosuka, Kanagawa, Japan; 2 Institute for Molecular and Cellular Regulation, Gunma University, Maebashi, Gunma, Japan; 3 Department of Chemistry, Tokyo Metropolitan University, Hachioji, Tokyo, Japan; Laboratoire de Biologie du Développement de Villefranche-sur-Mer, FRANCE

## Abstract

Many members of the tripartite motif (TRIM) family of ubiquitin ligases localize in spherical, membrane-free structures collectively referred to as cytoplasmic bodies (CBs) in a concentration-dependent manner. These CBs may function as aggresome precursors or storage compartments that segregate potentially harmful excess TRIM molecules from the cytosolic milieu. However, the manner in which TRIM proteins accumulate into CBs is unclear. In the present study, using TRIM32, TRIM5α and TRIM63 as examples, we demonstrated that CBs are in a liquid droplet state, resulting from liquid-liquid phase separation (LLPS). This finding is based on criteria that defines phase-separated structures, such as recovery after photobleaching, sensitivity to hexanediol, and the ability to undergo fusion. CB droplets, which contain cyan fluorescent protein (CFP)-fused TRIM32, were purified from HEK293 cells using a fluorescence-activated cell sorter and analyzed by LC-MS/MS. We found that in addition to TRIM32, these droplets contain a variety of endogenous proteins and enzymes including ubiquitin. Localization of ubiquitin within CBs was further verified by fluorescence microscopy. We also found that the activation of the intracellular ubiquitination cascade promotes the assembly of TRIM32 molecules into CBs, whereas inhibition causes suppression. Regulation is dependent on the intrinsic E3 ligase activity of TRIM32. Similar regulation by ubiquitination on the TRIM assembly was also observed with TRIM5α and TRIM63. Our findings provide a novel mechanical basis for the organization of CBs that couples compartmentalization through LLPS with ubiquitination.

## Introduction

Tripartite motif-containing (TRIM) proteins (also known RBCC proteins) represent a large family of ubiquitin E3 ligases consisting of an N-terminal RING finger, one or two B-boxes, and a predicted coiled-coil domain [1, 2]. The RBCC motif is usually followed by additional and variable C-terminal domains, such as SPRY, COS, or NHL, which play a role in substrate recognition and binding [1, 2]. More than 70 members of the TRIM family have been identified in the human genome and they are widely distributed among various tissues. A genetic analysis revealed that these TRIM members can be divided into at least two groups, based on distinct evolutionary properties [3]. Group 1 is more ancient than group 2 and has been implicated in basic functions. In contrast, group 2 is younger and more dynamic, possibly acting as a TRIM gene "reservoir" for the development of novel TRIM functions [3].

TRIM family proteins play key roles in a variety of physiological processes including cell proliferation, differentiation, development, and the immune response [4]. Thus, the cellular levels of TRIM proteins must be tightly controlled. Deregulated expression of TRIM proteins is linked extensively to the development and/or progression of various diseases including neuropsychiatric disorders, such as Alzheimer’s disease and schizophrenia, inflammatory diseases, infectious diseases, chromosomal abnormalities, and developmental diseases [5]. Aberrant expression of TRIM proteins has been reported as relevant biomarkers for several cancers. Surprisingly, TRIMs exhibiting the highest association with cancer include TRIM11, 14, 24, 25, 27, 28, 29, 33, 37, 44, and 59, and each is associated with multiple (at least five) cancers [6]. These findings suggest that the proper control of cellular TRIM concentration is important for the maintenance of normal cell physiology.

Many TRIM proteins are prone to aggregate and readily form spherical hollow inclusions known as cytoplasmic bodies (CBs) depending on their expression levels in cells [1]. In general, when TRIM proteins are expressed at low levels, their CBs are primarily distributed throughout the cytoplasm as small foci or dots. As expression increases, the small foci increase in size and fuse with one another to form larger cytoplasmic clusters, known as CBs, around the nucleus [1, 7–10]. Although the exact role of these CBs in TRIM function is not fully understood, recent studies, particularly with TRIM5α and TRIM32, have suggested that CBs may function as aggresome precursors or storage compartments of overexpressed TRIM polypeptides [10–13]. Because aberrant expression of TRIM proteins is closely associated with various diseases, sequestering potentially harmful excess TRIM molecules from the cytosolic milieu may be important for normal cell function. Nonetheless, little is known about the mechanism by which TRIM proteins accumulate into CBs.

Our previous work revealed that 14-3-3 proteins bind directly with TRIM32 when protein kinase A phosphorylates the latter at Ser651 [9]. This association suppresses the formation of TRIM32-containing CBs by retaining phosphorylated TRIM32 in the soluble cytosolic fraction [9]. We subsequently demonstrated that heat shock protein 70 (HSP70) has an opposite effect on 14-3-3s in concert with HSP40, in which the association facilitates CB formation by accumulating TRIM32 polypeptides into insoluble CBs, depending on intrinsic ATPase activity [10]. In the present study, we used TRIM63 and TRIM5α in addition to TRIM32 to further characterize the CB formation process. We found that these TRIM proteins undergo LLPS to form CBs that exhibit liquid properties in living cells. The LLPS-driven condensation of TRIM proteins in CBs is induced by ubiquitination, which suggests a novel mechanism for the organization of TRIM CBs that links cytoplasmic compartmentalization through LLPS with ubiquitination.

## Materials and methods

### Materials

The expression plasmids generated in this study are listed in S1 Table with plasmid names, vector names, primers and templates. The point mutants of the TRIM proteins were created using PCR-based targeted mutagenesis [9] and confirmed by DNA sequence analysis. Other plasmids used in this study are shown in Ref. [10], except for HttQ97-RFP [14], FLAG-TRIM5α [15] and FLAG-TRIM63 [16], which were kindly provided by Dr. Kaganovich (University Medical Center Goettingen) and Dr, Inoue (University of Tokyo), respectively. The expression plasmid, YFP-Ub, was purchased from Addgene, and the pECFP-C1 and pmCherry-C1 vectors were purchased from Clontech. Polyclonal anti-TRIM32 and monoclonal anti-tubulin antibodies were obtained from Santa Cruz Biotechnology and Sigma, respectively. 1,6-Hexanediol and MLN-7243 were obtained from Tokyo Kasei and Selleck, respectively.

### Cell culture

Cells were maintained in DMEM medium (Invitrogen Life Technologies) supplemented with 10% inactivated FBS (Cell Culture Bioscience), 0.5% (w/v) penicillin and streptomycin (Invitrogen) at 37°C in a 5% CO_2_ humidified atmosphere.

### Fluorescence microscopy

Cells were placed on a sterile micro cover glass (18 × 18 mm, Iwaki Co.) in 6-cm dish and incubated overnight. The cells were then transfected with 2 μg of expression plasmids using Lipofectamine 2000 (Invitrogen). After 24 hours, the micro cover glass was removed and the cells were washed with PBS (phosphate-buffered saline). The washed cells were then fixed with 4% paraformaldehyde phosphate buffer solution for 20 min, mounted with antifade reagents (SlowFade^Ⓡ^ Antifade Kit, Life Technologies) and visualized using a confocal fluorescence microscope (FluoView 10i, Olympus).

### Fluorescence recovery after photobleaching (FRAP) measurement

HEK293 cells were placed on 3.5-cm dishes and incubated overnight. The cells were then transfected with 2 μg of CFP-TRIM32, CFP-TRIM5α or CFP-TRIM63 using Lipofectamine 2000. After 24 hours, the cells were removed from the dishes with trypsin-EDTA (Invitrogen), transferred to 35-mm glass base dish (glass Φ12, Iwaki Co.), and incubated for 24 h to allow the cells to adhere. Images were acquired on an inverted confocal microscope (FluoView 100, Olympus) with a 100× oil immersion objective lens using the 488-nm line of an argon laser. The CB region of interest was selected and pre-processed images were collected prior to bleaching. Bleaching was done using FV10-ASW software (Olympus) with 100 repetitions at 100% laser intensity. After bleaching, images were captured periodically every 30 s for 5 min at a 25% laser intensity. Fluorescence recovery was measured by comparing the fluorescence observed at a point in time relative to the original fluorescence observed before bleaching. The fluorescence recovery rate, R (%), was calculated using a previously published method [17]: R (%) = [(Ft − Fb)/(Fpre − Fb)] × 100, where Ft is the average intensity of the bleached area after recovery at a given time, Fb is the fluorescence intensity outside the CB area (background), and Fpre is the fluorescence intensity before photofading.

### Cell fusion experiment

HEK293 cells expressing CFP-TRIM32, CFP-TRIM5α or CFP-TRIM63 were sorted with a cell sorter (SH800S, SONY) as described [18]. The sorted cells were mixed with HEK293 cells expressing mCherry-TRIM32, mCherry-TRIM5α, and mCherry-TRIM63, and fused using a cell fusion reagent (GenomeONE^TM-^CF, Ishihara Sangyo Co.) according to the manufacturer’s instructions and incubated at 37°C on micro cover glasses in 6-cm dishes to adhere for 6 hours. After incubation, the cells were fixed and visualized with a confocal fluorescence microscope using CFP and/or mCherry filters.

### Isolation of TRIM32 CBs from HEK293 cells

HEK293 cells (grown in a 10-cm dish) were transfected with 5 μg of CFP-TRIM32 plasmid using a calcium phosphate method. After 24 hours, the cells were kept on ice and washed three times with PBS. The cells were then lysed in 500 μL of lysis buffer [9] [50-mM Tris-HCl, pH7.5, 150-mM NaCl, 10% (w/v) glycerol, 100-mM NaF, 10-mM EGTA, 1-mM Na_3_VO_4_, 1% (w/v) Triton X-100, 5-μM ZnCl_2_, and 1.0-μL Protease Inhibitor Cocktail (Calbiochem)] and collected in 1.5-ml microfuge tubes. The cells were lysed using a sonicator (AS ONE) for 2 min and agitated for 1 min (repeated three times). The cell lysates were then filtered with a cell strainer (5-mL Polystyrene Round-Bottom Tube with Cell-Strainer Cap, FALCON). The samples were analyzed in a cell sorter equipped with 405 and 488 nm lasers (SH800S, SONY). TRIM32-containing CBs were sorted using three gate filters (mCFP, FSC and BSC) and collected in 0.5 mL cold PBS. The mCFP filter was gated to exclude particles from untransfected cells. The FSC and BSC filters were gated for particles larger than 1.5 μm in size based on a previous estimation using size reference beads. Of the sorted particles, a 10,000-particle aliquot (representing ~0.16% of starting 6,000,000-events) were collected in a 1.5-mL microfuge tube and solubilized with 50 μL of ionic liquid (IL, 1-Butyl-3-methylimidazolium thiocyanate) − 0.5-M NaOH mixture (40% v/v IL) (hereafter *i*-soln) for complete solubilization of the aggregated proteins [19]. A control experiment was performed in parallel as mentioned above with naive HEK293 cells (transfected with liposome alone), and a 15-particle aliquot (representing ~0.0002% of equal 6,000,000-events of starting particles) were solubilized with *i*-soln.

### LC-MS/MS analysis

The CB and control samples dissolved in *i*-soln were processed using a microbead-based and organic-media-assisted proteolysis strategy (*i*BOPs), essentially according to previously described procedures [19, 20]. Briefly, solubilized proteins were adsorbed onto POROS R2 microbeads (diameter 50 μm, PerSeptive Biosystems, Inc.), and the beads were washed sequentially with 100 μL of acetone, 100-mM Tris-HCl, pH 8, and water. Trypsin digestion was conducted with 0.5 μg trypsin in 10 μL of 5-mM Tris-HCl buffer (pH 8.8) and 60% CH_3_CN at 37°C overnight with rotation. The digested samples were analyzed in a nanoscale LC-MS/MS system with a quadrupole-orbitrap hybrid mass spectrometer (Q Exactive; Thermo Fisher Scientific). The MS/MS data were converted into Mascot-compatible format with Proteome Discoverer (Thermo Fisher Scientific) as previously described [20]. A database search was performed in MASCOT v. 2.4.0 (Matrix Science K.K.) against the UniProt human protein database. Peptide identification criteria were based on vendor definitions (P < 0.05; Matrix Science K.K.). Identification was also manually confirmed by inspecting the MS/MS spectra. The relative abundance change in the CB sample was determined by comparing the relative abundance factor (RAF) in the CB sample with the RAF in the control sample and expressed as a ratio (RAF CB sample/RAF control sample). The RAF values were calculated by dividing the number of peptide identifications for the protein by the molecular weight of the protein as described previously [21].

### 1,6-Hexanediol treatment

HeLa cells were transfected with plasmids. After 24 hours, the cell culture medium was replaced with 10% (w/v) 1,6-hexandiol (dissolved in the cell culture medium without penicillin and streptomycin) and culturing continued for an additional 12 h.

### MLN-7243 treatment

Hek293 cells were transfected with plasmids. After 4 hours, the cell culture medium was replaced and 5-μM MLN-7243 (dissolved in DMSO) were added. Culturing then continued for another 20 h.

### Cell counting and statistical analysis

The number of cells in which CFP-TRIM protein expression was confirmed by microscopy was used as the population, and cells containing CBs or mutant bodies with a spherical shape of 1 μm or larger were counted as CB-bearing cells and characterized as described previously [10]. Statistical analyses of the number of CB-bearing cells were performed using Microsoft Excel. Graphical plots were also made in Excel. Data were expressed as means ± SD. The number of cells counted is indicated in the figure legends.

## Results and discussion

### TRIM CBs are in a liquid droplet state produced by LLPS

**Fig 1:**
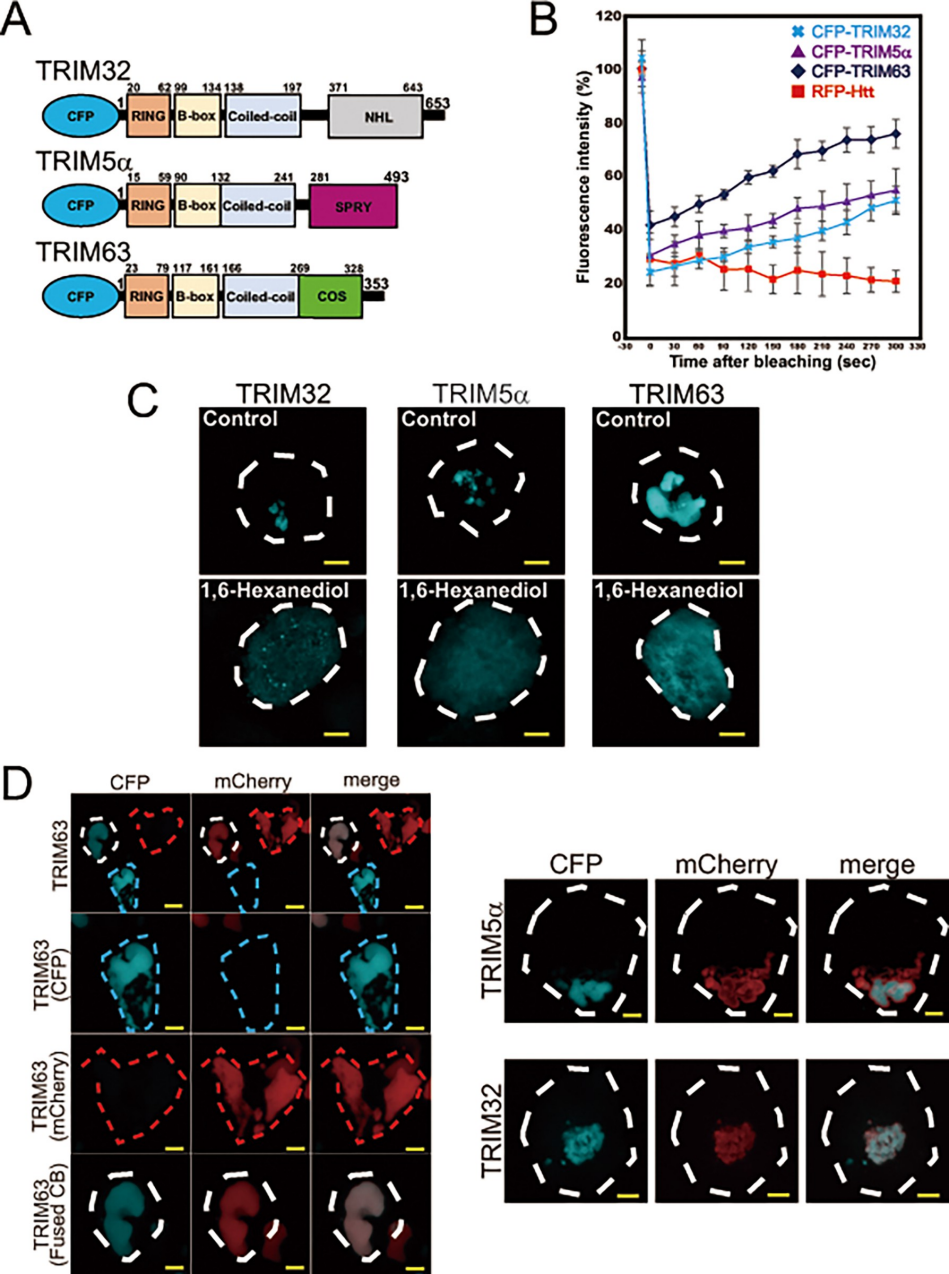
TRIM CBs are liquid droplets resulting from LLPS. (**A**) Schematic representation of CFP-tagged human TRIM32, TRIM5α and TRIM63 proteins. The numbers in the figure indicate the amino acid numbers in the primary sequence of each TRIM species. (**B**) Analysis of the liquid property of TRIM CBs by FLAP analysis. HEK293 cells were transiently transfected with CFP-TRIM32, CFP-TRIM5α, CFP-TRIM63, or RFP-Htt. After 48 h, the CBs or Htt inclusions generated in these cells were analyzed by FRAP. Graphs indicate the average recovery over time from five experiments. Error bars represent the standard error. (**C**) Analysis of the sensitivity of TRIM CBs to 1,6-hexanediol. HeLa cells expressing CFP-TRIM32, CFP-TRIM5α, or CFP-TRIM63 were incubated with or without 1,6-hexanediol for 12 h and visualized with fluorescence microscopy. The dotted lines indicate the cell outlines. Bars, 5 μm. (**D**) Analysis of the fusion properties of TRIM CBs. (Left panels) HEK293 cells were expressed with CFP-TRIM63 or mCherry-TRIM63. The cells expressing respective florescent proteins were fused and observed under a fluorescence microscope. Top panel is a representative fluorescence cell image observed using the CFP and mCherry filters. The second, third, and bottom panels are magnified images of the three cells shown in the top panel; cells with only cyan-colored (CFP) and red-colored (mCherry) CBs (second and third panels, respectively) and cells with fusion of two colored CBs within the same cells (bottom panel, merged). (Right panels) HEK293 cells expressing CFP-TRIM5α or mCherry-TRIM5α (upper panels) and CFP-TRIM32 or mCherry-TRIM32 (lower panels) and analyzed as above. The representative cell images with fused CBs are shown. Bars, 5 μm.

A previous microscopic study showed that CBs containing TRIM5α have highly dynamic structures that can readily undergo fission and fusion [8]. Because such properties of TRIM5α CBs are reminiscent of protein droplets formed by LLPS [22, 23], we assumed that TRIM-containing CBs also have liquid-like characteristics. To test this assumption, we fluorescently-labeled TRIM5α as well as TRIM32 and TRIM63 by conjugating CFP (Fig 1A). The TRIM members used in this study belong to different groups of the TRIM family: TRIM5α from group 2 and TRIM32 and TRIM63 from group 1 [3].

Initially, we analyzed the mobility of TRIM molecules within CBs using FRAP. We expressed CFP-TRIM32, CFP-TRIM5α, and CFP-TRIM63 in HEK293 cells to generate CBs, bleached the respective CB regions using a high-powered laser, and reordered subsequent CFP fluorescence. As shown in Fig 1B, the CFP signals were all recovered over time. In contrast, no recovery of the fluorescent signal was observed for HttQ97-RFP, an amyloidogenic protein known to be sent to IPOD (insoluble protein precipitate) (Fig 1B). This is consistent with the previous data showing that IPOD is completely immobile [14]. These results suggest that TRIM-containing CBs have mobile structures.

1,6-Hexanediol is an aliphatic alcohol known to destabilize certain protein droplets that arise from LLPS (e.g., Rad52 foci [24], proteasome foci [25], etc.). We determined whether this alcohol can also destabilize TRIM CBs once generated in HeLa cells. As shown in Fig 1C, the addition of hexanediol disassembled all TRIM CBs tested but not with vehicle (DMSO) alone, suggesting that TRIM CB structures are sensitive to this droplet-disrupting agent. Importantly, destabilization of the CBs with hexanediol treatment was initially detected within 2 hours; however, the complete disassembly of CBs (Fig 1C) required longer exposure times (~12 h). The long incubation period required for disrupting CB structures suggests that hydrophobic interactions may not be the sole basis for CB stability, which has been reported for stress granules [26].

We also tested whether CBs can fuse with one other in living cells. We first used TRIM63 because it has the largest CB size among the three TRIM proteins examined in the present study. After transfecting CFP-TRIM63 into HEK293 cells to generate cyan-colored CBs, we isolated TRIM-expressing cells using a fluorescence-activated cell sorter. Next, we created a new TRIM63 construct fused with mCherry at its N-terminus instead of CFP, and the mChery-TRIM63 was transfected into HEK293 cells to generate red-colored CBs. After mixing these two lines of expressing cells, they were fused using a cell fusion kit and the state of the CBs was monitored under a fluorescence microscope after 6 h. The results indicated that while either cyan- or red-colored CBs were observed in many cells (Fig 1D; left, top panel), >5% of the cells exhibited bicolored CBs, indicating that the CBs had fused within the cells (Fig 1D; left, bottom panel, see ‘merge’). Similarly, bicolored CBs were also observed in experiments with TRIM5α and TRIM32 (Fig 1D, right panels). This suggests that all CBs containing TRIM32, TRIM5α, and TRIM63 can undergo fusion in living cells.

All of the properties of CBs described above were consistent with those reported that define liquid droplets arising from LLPS [22, 23]. These results indicate that TRIM-containing CBs are likely in a liquid droplet state and formed by LLPS.

### Isolation and proteomic characterization of TRIM32-containing CBs

**Fig 2:**
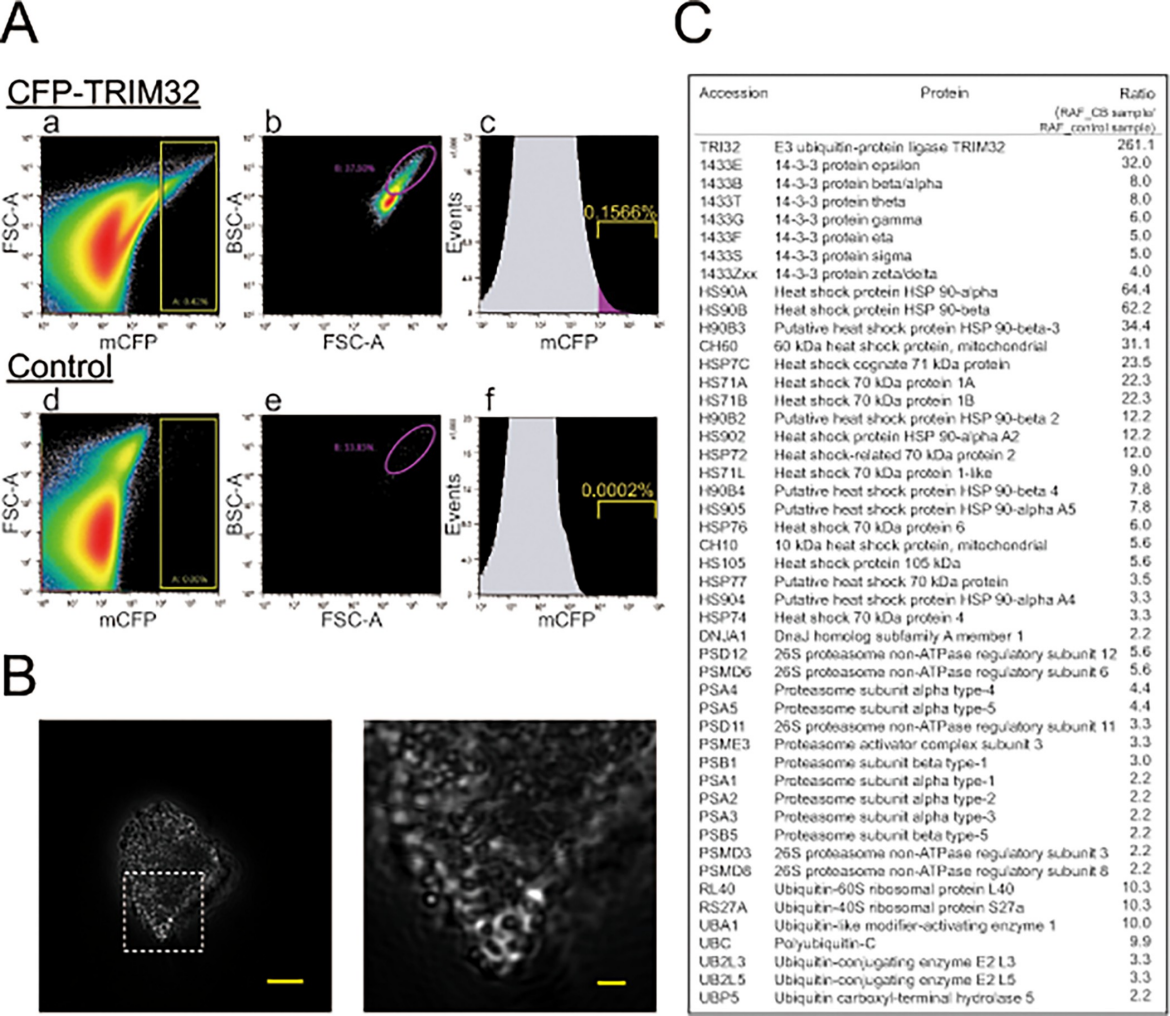
Proteomic characterization of proteins associated with TRIM32 CBs isolated from HEK293 cells. (A) Purification of TRIM32-contaiining CBs using a cell sorter. HEK293 cells were transiently transfected with (upper) or without (lower) CFP-TRIM32, lysed in 1% Triton X100, and the cell lysates were analyzed with a cell sorter to sort particles with the CFP filter (a,d) and then with FSC and BSC filters (b,e). Numbers represent the relative frequency of finally sorted particles against total particles (expressed as percentages; c,f). (B) Phase contrast microscopy image of purified CB clumps. (b) the magnitude image of the box region shown in (a). Bars indicate 5 μm (a) and 1 μm (b), respectively. (C) List of CB-associated proteins involved in protein quality control. The relative abundance factor (RAF) of the CB sample was compared with the RAF of the control sample to obtain a ratio (RAF_CB sample/RAF_control sample). The method for calculating RAF values is described under Materials and Methods.

To elucidate the mechanism controlling the formation of CB droplets at the molecular level, we isolated CBs from HEK293 cells using a cell sorter. Of the CBs containing CFP-TRIM32, -TRIM5α, or -TRIM63, we selected CFP-TRIM32 CBs, because they were the most stable in our cell lysis buffer containing 1% Triton X-100. In the scattergram for the flow cytometric analysis of the CFP-TRIM32 lysate, (Fig 2A, upper panels), numerous microparticles with high fluorescent signals were observed [Fig 2A(a)]. In contrast, fewer such fluorescence particles were detected in the control cell lysate not-expressing CFP-TRIM32 [Fig 2A(d)], indicating that the particles are specific. Regions were then created on the histogram [Fig 2A(a), indicated by yellow box] and microparticles with sufficient fluorescence intensity within this region were sorted. The sorted particles were further separated by size greater than 1.5 μm [Fig 2A(b), indicated by purple circle] and a final CB fraction was obtained. Of the total number of particles, approximately 0.16% were found in this final fraction. For the control cells, the number of particles in the same region was only 0.0002%, which indicates that the final CB preparation is highly pure [Fig 2A(c), (f)]. High-resolution phase contact microscopy revealed that the final preparation contained crowded CB clumps that were partially fused or associated with one another (Fig 2B).

Of the sorted CB particles, 10,000 particles were solubilized with *i*-soln, which is a mixture of ionic liquid and alkaline aqueous solution [19], processed with trypsin using the *i*BOPs method [19], and analyzed by LC-MS/MS. We identified 483 cytoplasmic proteins that displayed at least a two-fold enrichment in the CFP-TRIM32 preparation compared with the control preparation as CB-associated proteins (Ratio >2, S2 Table). Of these proteins, HSP70 and HSP40, and a series of 14-3-3 protein isoforms were previously characterized (Fig 2C). Four reported TRIM32 substrates, actin, actinin, tropomyosin, desmin [27, 28], were also included in these proteins (S2 Table). In addition, they contained many components of the protein quality control machinery, such as proteasome subunits and ubiquitin, whose relevance to TRIM32 CBs has not yet been fully defined (Fig 2C). These results suggest that the CB droplets are heterogeneous protein mixtures that contain many cytosolic proteins other than TRIM32.

### Ubiquitin is a common component of CB droplets containing TRIM32, TRIM5α, and TRIM63

**Fig 3:**
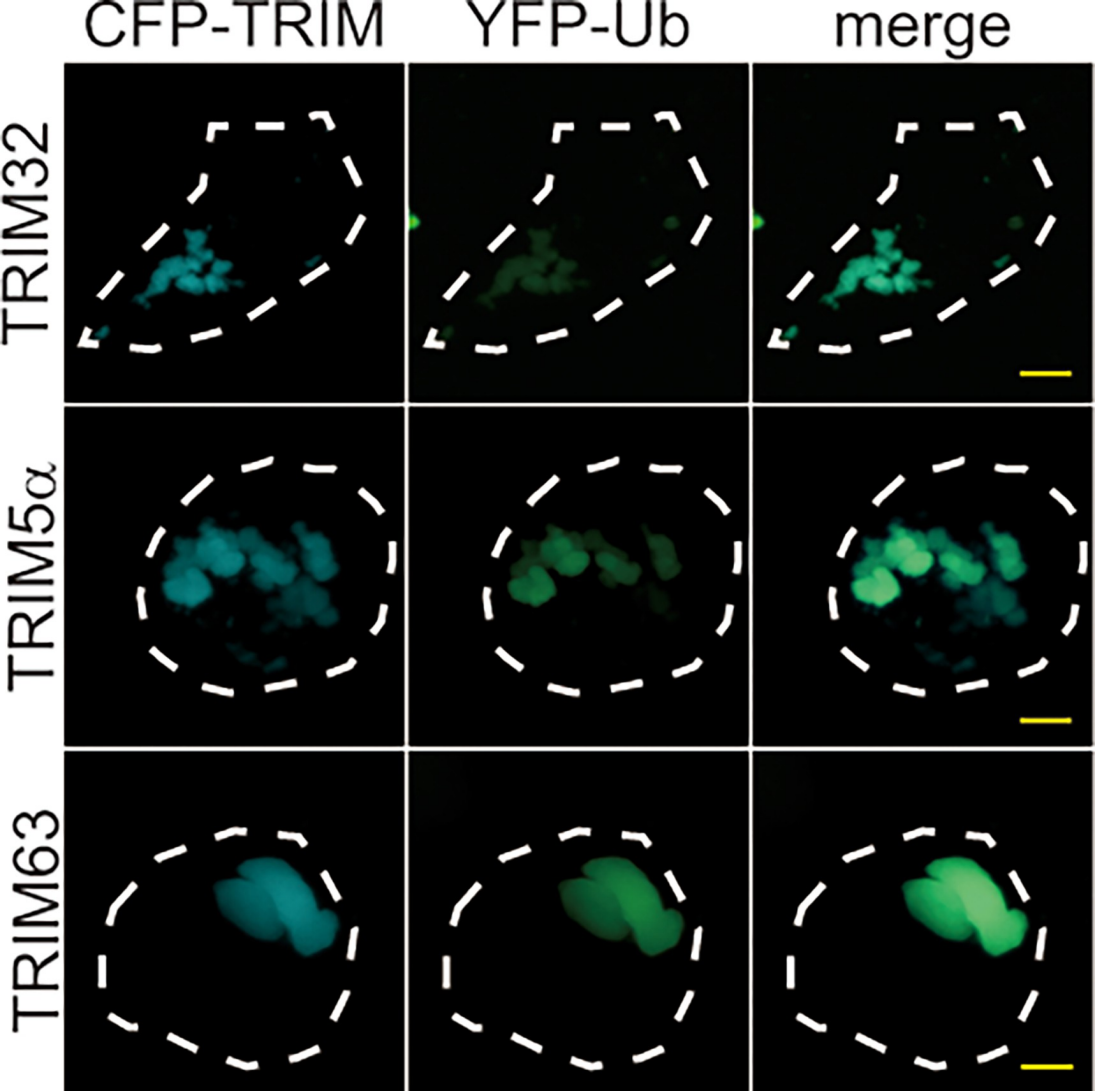
Localization of ubiquitin in TRIM CBs. HEK293 cells were co-transfected with CFP-TRIM32, CFP-TRIM5α, or CFP-TRIM63, and YFP-ubiquitin (YFP-Ub) and visualized by fluorescence microscopy. Representative images of the cells from at least three independent experiments are shown. Bars, 5 μm.

Ubiquitin (or ubiquitination) regulates the formation and dynamics of protein droplets, including p62 bodies [29], proteasome foci [25], and ubiquilin-2 bodies [30]. Because our MS analysis identified ubiquitin as a potential component of TRIM32-containing CBs, we confirmed that ubiquitin is actually included in CBs. Therefore, we co-expressed CFP-TRIM32 and YFP-ubiquitin in HEK293 cells and analyzed their localization by fluorescence microscopy. The analysis revealed that the fluorescence signals of CFP-TRIM32 CBs and YFP-ubiquitin completely overlapped within the same cells (Fig 3, top three panels). This colocalization validated our MS result, and strongly suggests that ubiquitin is indeed included in CB droplets. Next, we analyzed whether YFP-ubiquitin also localizes with CB droplets containing CFP-TRIM5α and CFP-TRIM63 with a similar assay as described above and confirmed that YFP-ubiquitin also colocalized with both of these CBs (Fig 3, second and bottom panels). The colocalization images observed with CFP-TRIM CBs and YFP-ubiquitin appeared to be specific because coexpression of CFP alone and YFP-ubiquitin did not produce any aggregation and body formation (S1 Fig). Together, these observations suggest that ubiquitin is a common component of CB droplets, which contain TRIM32, TRIM5α, and TRIM63.

### Ubiquitination promotes the LLPS-driven assembly of TRIM32 in CBs

**Fig 4:**
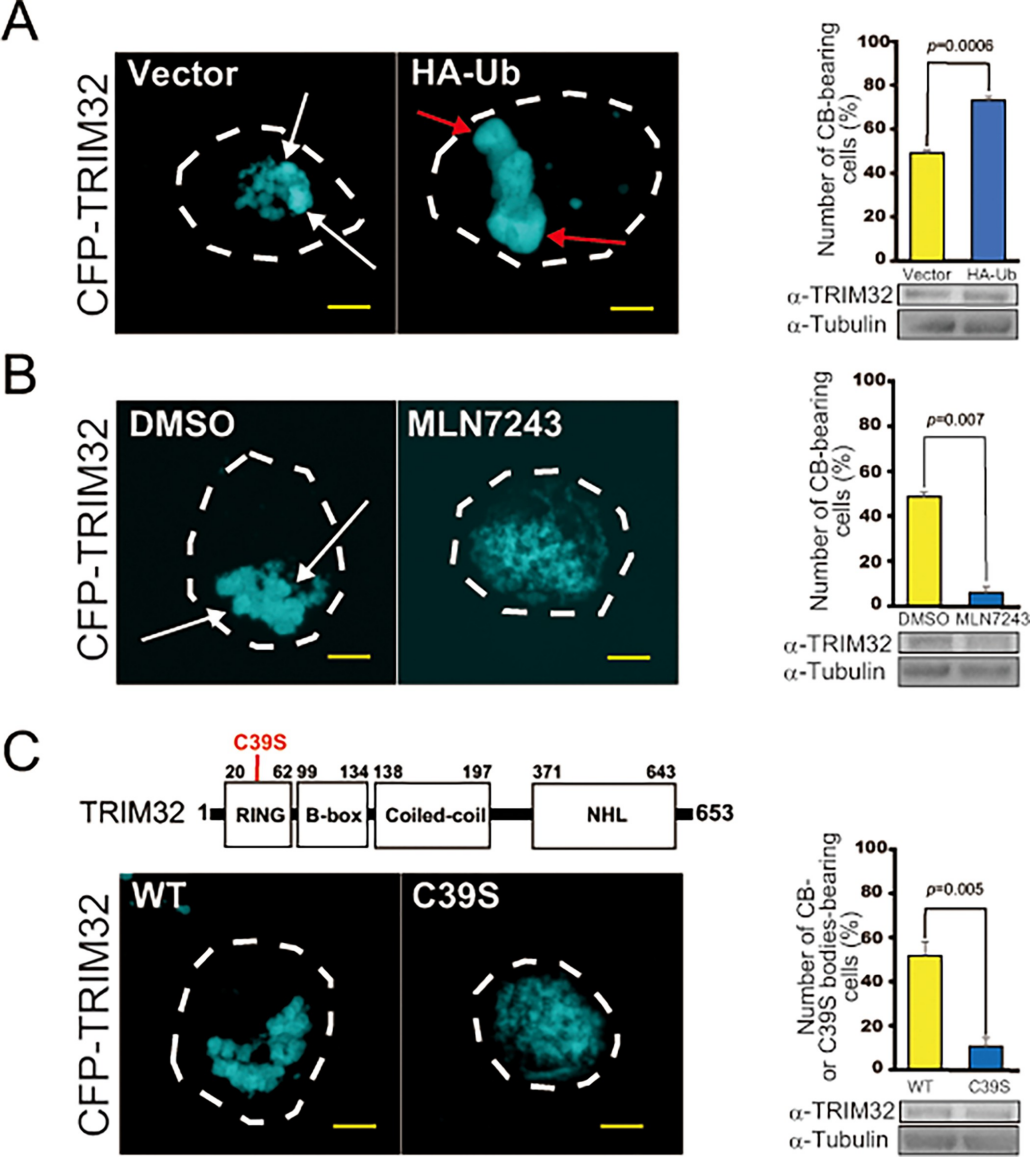
Ubiquitination promotes the accumulation of TRIM32 molecules into CB droplets. (**A**) HEK293 cells were co-transfected with CFP-TRIM32 and vector alone or HA-ubiquitin (HA-Ub) and visualized with fluorescence microscopy. (Left) Representative images of cells from at least three independent experiments are shown. Dotted lines indicate the cell outlines, white arrows indicate normal CBs, and red arrows indicate giant CBs. (Right, upper) The number of CB-bearing HEK293 cells were quantified from 40–60 cells in three experimental repeats (*n* = 3). Data are means ± SD. *p* indicates *t*-test. (Right, lower) Whole proteins from HEK293 cells were extracted with 1% SDS after 24 hours of transfection and immunoblotted with anti-TRIM32 and anti-tubulin. Tubulin was used as an internal control. (**B**) CFP-TRIM32 transfected HEK293 cells were exposed to DMSO or 5 μM MLN-7242 dissolved in DMSO and analyzed as in (**A**). *n* = 2. (**C**) HEK293 cells were transfected with CFP-TRIM32 or its C39S point mutant and analyzed as in (**A**). *n* = 3. Bars, 5 μm.

Having confirmed that ubiquitin is indeed localized in TRIM32 CBs, we determined whether ubiquitination affects the CB formation pathway mediated by LLPS. Initially, we overexpressed HA-tagged ubiquitin in HEK293 cells to activate the intracellular ubiquitination cascade and evaluated its effect on CB droplet formation. As shown in Fig 4A, co-expression of HA-ubiquitin with CFP-TRIM32 produced CBs that were larger in size and volume compared with those generated by vector alone. This suggests that overexpression of ubiquitin promoted the accumulation of TRIM32 molecules into CB droplets (see Fig 4A, red arrows). Indeed, when HA-ubiquitin was co-expressed, 80% of the CFP-TRIM32-expressing cells contained CBs larger than 1 μm in size by fluorescence microscopy compared with ≤50% of cells transfecting with vector alone (Fig 4A, right). We next examined the effect of inhibiting the ubiquitination cascade with MLN-7243, an inhibitor of ubiquitin-activating enzyme (UAE1) [25]. Contrary to the result obtained by HA-ubiquitin overexpression, the addition of MLN-7243 produced CBs that were smaller in size and volume compared with vehicle alone (Fig 4B, left). The number of cells bearing CBs at >1 μm in size was less than 10% (Fig 4B, right), suggesting that MLN-7243 suppressed the accumulation of TRIM32 into CBs. Importantly, similar effect of suppressing the CB formation pathway with MLN7243 was also observed even when HA-ubiquitin was overexpressed (S2 Fig), supporting a role of ubiquitination in TRIM32-generated CBs. In both these experiments, we confirmed that the morphological changes observed for TRIM32 CBs were not the result of an alteration in total levels of expressed TRIM32 proteins, because nearly equal amounts of TRIM32 protein were detected in each set of experiments by comparative immunoblotting (Fig 4A and 4B, right bottom panels). Therefore, we concluded that ubiquitination acts as a positive regulator in the CB formation pathway by LLPS.

To determine whether the stimulatory effects observed for CB formation by ubiquitination are responsible for the E3 ligase activity of TRIM32 or the activity of other E3 ligases endogenously expressed in HEK293, we generated a CFP-TRIM32 C39S mutant (Fig 4C, top). The C39S mutant contains a point mutation in which the second Cys residue (39th residue from the N-terminus) in the RING domain of TRIM32 is replaced by a Ser residue, resulting in loss of intrinsic E3 ligase activity [31, 32]. The overexpressed C39S variants were similarly assembled into insoluble bodies as wild-type TRIM32; however, the C39S bodies were smaller in size compared with that of TRIM32 CBs (Fig 4C, bottom, left). Also, the number of cells bearing C39S bodies with a size >1 μm was only ~10% (Fig 4C, bottom right). These properties of C39S bodies were very similar to that observed for MLN7243-treated TRIM32 CBs (see Fig 4B). Thus, the E3 ligase activity of TRIM32 may be responsible for LLPS-driven CB formation promoted by the ubiquitination reaction.

### Ubiquitination also promotes the formation of CB droplets containing TRIM5α and TRIM63

**Fig 5:**
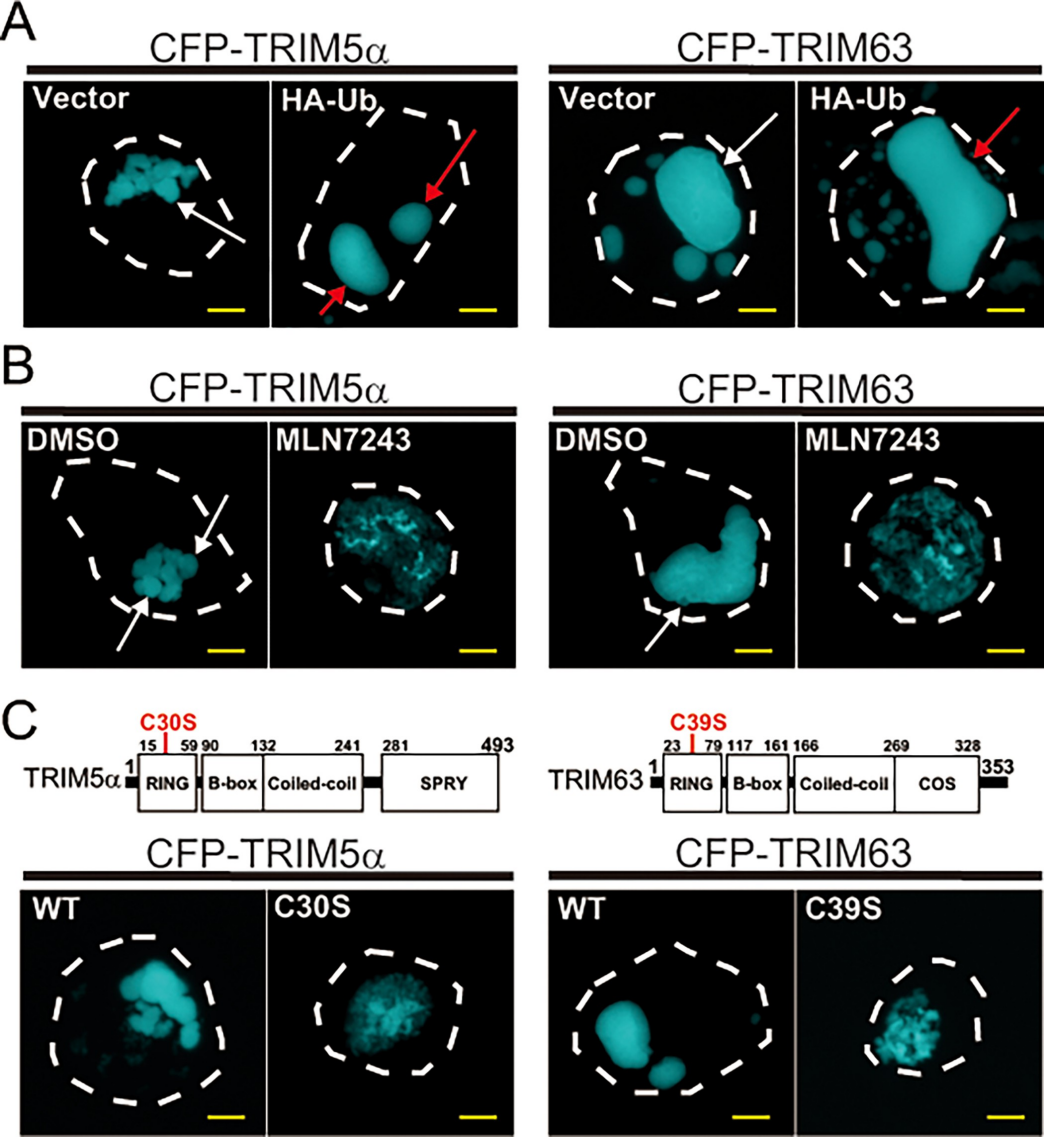
Ubiquitination promotes the formation of CB droplets containing TRIM5α and TRIM63. (**A**) Fluorescence microscopy images of HEK293 cells transfected with CFP-TRIM5α together with or without HA-ubiquitin (HA-Ub, left two panels) or with CFP-TRIM63 together with or without HA-Ub, (right two panels). (**B**) Fluorescence microscopy images of HEK293 cells containing CFP-TRIM5α and CFP-TRIM63 CBs incubated in the presence or absence of 5 μM MLN-7243. **(C)** Fluorescence microscopy images of HEK293 cells transfected with CFP-TRIM5α and its C30S mutant or with CFP-TRIM63 and its C39S mutant. Bars, 5 μm.

Having found that ubiquitin is commonly included in not only TRIM32, but also TRIM5α and TRIM63 CBs (Fig 3), we examined whether ubiquitination regulates the formation of CB droplets containing TRIM5α and TRIM63. As shown in Fig 5A and 5B, both experiments using HA-ubiquitin and MLN-7243 indicated that just like TRIM32, ubiquitination increased the accumulation of both TRIM5α and TRIM63 molecules into CB droplets. We also prepared CFP-TRIM5αC30S and CFP-TRIM63C39S variants by replacing the second Cys with Ser in the RING domain (corresponding to the identical position of Cys residue of the TRIM32C39S) and found that both mutants exhibited a defect in body formation as observed with TRIM32C39S (Fig 5C). Thus, ubiquitination appears to be a common mechanism by which controls CB droplet formation for all three TRIM proteins.

### Effect of mutation of reported TRIM32 self-ubiquitination sites on the CB droplet formation

The results described above indicate that ubiquitination is an important regulator of the CB formation process; however, it is unclear how this reaction drives TRIM assembly into CB droplets. In a previous study, Locke et al [33] demonstrated that the point mutant, TRIM32 D487N, in which Asp 487 (D) of TRIM32, the causative gene of limb-girdling muscular dystrophy type 2H (LGMD2H), was altered to contain an Asn residue (N), which does not undergo self-ubiquitination and CB formation. We have recently found that the PKA-mediated binding of TRIM32 to 14-3-3 impaired the ability of TRIM32 to self-ubiquitinate and localize into CBs [9]. These findings suggest that TRIM32 self-ubiquitination may act as a mechanism to control the accumulation of the protein into CB droplets.

**Fig 6:**
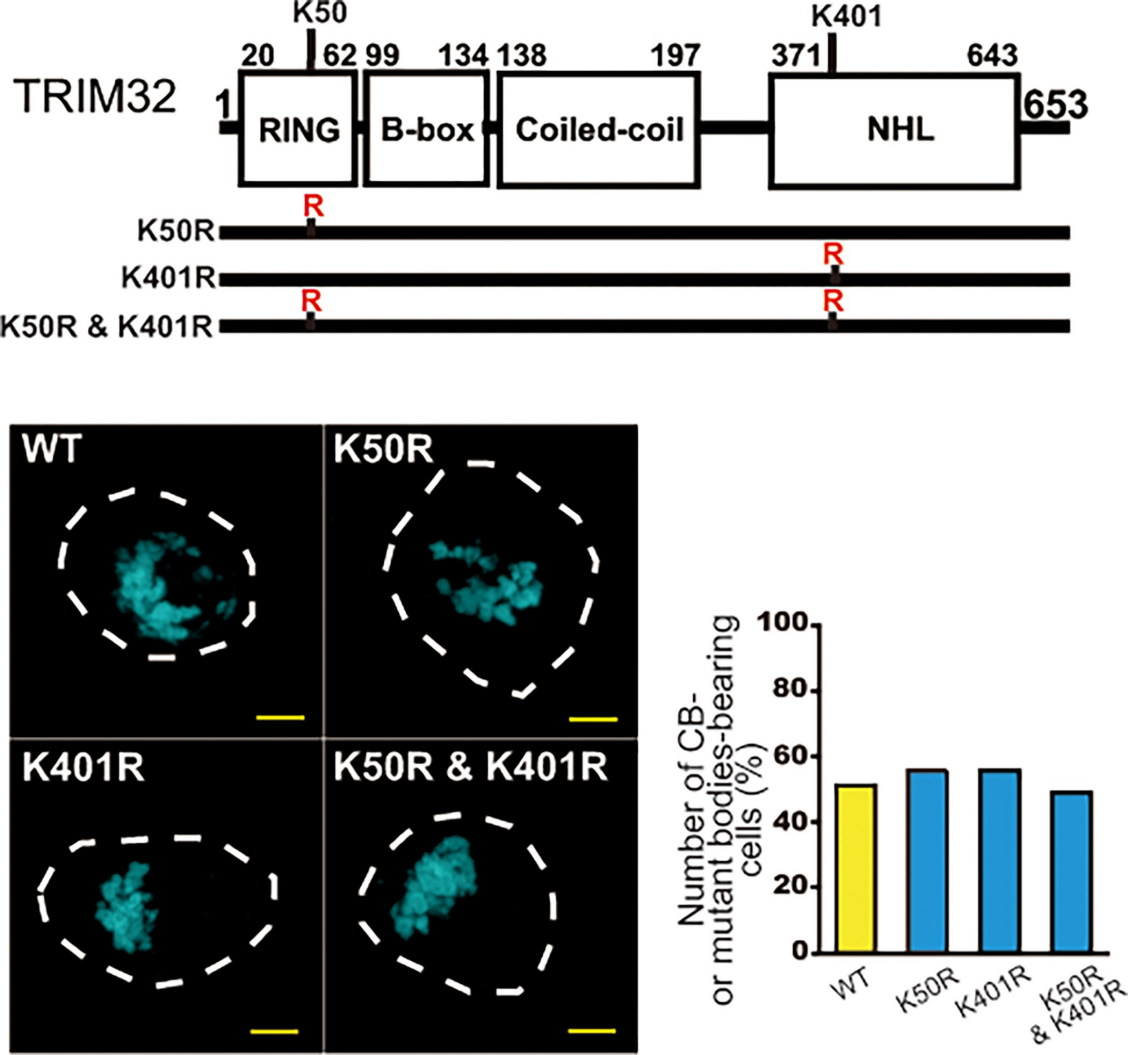
Effect of the TRIM32 K50R and/or K401R mutation on CB formation. The experiment was conducted as in Fig 4C, except the K50R and/or K401R mutants of CFP–TRIM32 were used instead of the C39S variant. *n* = 1. Bars, 5 μm.

Recently, Garcia-Garcia et al. [34] reported that TRIM32 is self-ubiquitinated at K50 and K40. To determine the effect of mutating these Lys residues on CB formation, we generated TRIM32K50R and TRIM32K401R mutants by replacing K (Lys) with R (Arg) and expressing them in HEK293 cells. However, less difference on CB formation was detectable between these mutants and wild-type TRIM32 by fluorescence microscopy (Fig 6). We also introduced double mutation on both of these self-ubiquitination sites, TRIM32K50R and K401R, and evaluated them as described above. Again, this double mutant did not result in any detectable difference compared with the wild-type TRIM32 upon CB assembly (Fig 6), suggesting that neither of the self-ubiquitination reactions at K50 and K401 affects CB formation, at least with any significance. These observations suggest that self-ubiquitination sites other than K50 and K401 may contribute to the control of CB droplet formation, but this will require further study.

Protein droplet formation by LLPS is considered a key physiological process that regulates various cell functions [22, 23]. In general, droplet formation occurs in response to environmental stresses such as heat shock, oxidative stress, osmotic shock, or increased protein expression [23, 35]. For example, stress granules (SGs) are formed in response to heat shock and hypoxia by scaffolding mRNPs, which is a complex of mRNA and RNA-binding proteins. SGs are thought to play a role in preventing the accumulation of abnormally folded proteins within the cell by temporarily arresting protein translation at the mRNA level, thereby protecting cells from further damage [36]. Proteasome foci (PFs) are formed in response to hyperosmotic stimuli by scaffolding RAD23B, a protein with a ubiquitin-binding domain, and ubiquitinated proteins. PFs play a cleansing role for damaged proteins by providing a site for protein degradation, thereby protecting cells during osmotic stress [25]. In the present study, we found that TRIM-containing CBs are also liquid droplets produced by LLPS. This suggests that CBs may locally sequester and preserve overexpressed TRIM molecules possibly as inactive forms. The formation of CB droplets is actively controlled by ubiquitination. This suggests that CB formation is a dynamic physiological event coupled with the cellular posttranslational network, rather than representing merely a physical phenomenon that depends upon the thermodynamic advantage of separating the high- and low-concentration phases. In fact, proper control of TRIM expression is essential for maintaining normal cell physiology, because abnormal TRIM expression is linked to the development of various diseases, such as cancer and muscular dystrophy [4, 5]. Therefore, we propose that CB droplet formation via LLPS is a type of defense mechanism that maintains cellular proteostasis during the stress of protein overexpression.

Theoretically, any overexpressed proteins that are prone to aggregate may trigger protein degradation pathways and be ubiquitinated. This suggests that the formation of CB droplets is merely an intermediate step in the ubiquitination-mediated clearance of overexpressed TRIM molecules. However, in the present study, we found that ubiquitination promotes the incorporation of overexpressed TRIMs into CBs, as demonstrated in Figs 4 and 5. We propose, therefore, that CBs are not only a reservoir for TRIM proteins destined for degradation but also act as a sorting center to segregate overexpressed, potentially harmful TRIM species from the cytosolic milieu. In the case of the p62 [29] and proteasome foci formation [25], polyubiquitin chains or ubiquitinated proteins have been shown to act as key players to drive LLPS. However, it is currently unknown whether the TRIMs directly scaffold the CBs or they are encapsulated into existing CBs induced by ubiquitination.

Recently, it was demonstrated that many TRIM proteins, including TRIM32, are involved in autophagy as autophagic regulators and receptors [37]. Consistent with these findings, certain TRIM32 bodies generated in tetracycline-inducible Flp-In T-Rex 293 cells colocalized with the autophagy marker proteins, LC3B and p62, which represent autophagosomes [12]. However, our LC-MS/MS analysis did not identify any LC3B or p62 molecules in the purified CB fraction (S2 Table). In addition, none of the other autophagy factors such as NBR1, NDP52, TAX1BP1, and OPTN [38–40], which had been shown to interact with TRIM32 by immunoprecipitation, were detectable in the analysis. During the LC-MS/MS analysis, we adjusted the cell sorter conditions to recover relatively large sized CBs (>1.5 μm). Therefore, although further analyses focusing on smaller sized CBs are needed, the present discrepancy between our results and that of Ref. [12] may result from the TRIM32 bodies purified in this study representing different entities than those observed in Ref. [12]. The heterogeneity of TRIM CBs has been established [11, 13, 41]. For example, stable expression of TRIM5α in cells results in the formation of small CBs with anti-HIV retroviral activity (ability to inhibit infection), whereas transient overexpression results in the formation of large inactive CBs with no inhibitory effects [41]. The formation of small CBs containing activated TRIM5α is promoted when the concentration level of TRIM5α in the cell is low, whereas a large number of giant CBs containing inactivated TRIM5α appear when the concentration of TRIM5α in the cell exceeds a specific level [11]. Therefore, the present findings indicate that there are at least two populations of CBs with different properties in a cell. This suggests that the component composition of these CB populations may not be identical.

In conclusion, we demonstrate for the first time that CBs containing three TRIM ubiquitin ligases (TRIM32, TRIM5α, and TRIM63) are all in a liquid droplet state arising from LLPS. We also demonstrate that the formation of these droplets is positively regulated by ubiquitination, which is responsible for auto E3 ligase activity. The three TRIM species employed in this study were selected because they are representative of both TRIM group 1 and 2 of the TRIM family [3]. Moreover, their substrate-binding domains (NHL, SPRY, COS) are also conserved even in other TRIM members. The results described here suggest a common mechanism for the organization of CBs applicable to many members of the TRIM family. Because deregulated expression of TRIM proteins contributes to various diseases, a better understanding of CB formation and its relationship to various diseases will be beneficial toward the design of new therapies for TRIM-associated diseases.

## Supporting information

S1 Table List of plasmids created in this study. (PDF)

S2 Table List of proteins associated with TRIM32-containing CBs identified by LC-MS/MS. (PDF)

S1 Fig Fluorescence images of HEK293 cells co-expressing CFP and YFP-ubiquitin. HEK293 cells were co-transfected with CFP and YFP-ubiquitin (YFP-Ub) and visualized by fluorescence microscopy as in Fig 3. Bars, 5 μm.
(TIF)

S2 Fig MLN7243 suppresses the formation of CB droplets even when HA-ubiquitin is overexpressed. HEK293 cells co-transfected with CFP-TRIM32 and HA-ubiquitin were exposed to DMSO or 5 μM MLN-7242 dissolved in DMSO and analyzed as in Fig 4B. *n* = 1. Bars, 5 μm.
(TIF)

S1 Raw images (PDF)
