# Supplementary material for: Ubiquitination-coupled liquid phase separation regulates the accumulation of the TRIM family of ubiquitin ligases into cytoplasmic bodies
Source: PLoS One. 2022 Aug 5;17(8):e0272700. doi: 10.1371/journal.pone.0272700 (PMC9355226; doi:10.1371/journal.pone.0272700)
Supplement: S1 Table — (PDF) [file pone.0272700.s001.pdf]

S1 Table List of plasmids created in this study

Plasmids	Vectors	Primers	Templates
CFP-TRIM32	pECFP-C1	Fw: 5'-cgggatccatggctgcagcagcagcttc-3'<br>Rv: 5'-cggaattcctatggggtggaatatcttc-3'	FLAG-TRIM32
mCherry-TRIM32	pmCherry-C1	Fw: 5'-cgggatccatggctgcagcagcagcttc-3'<br>Rv: 5'-cggaattcctatggggtggaatatcttc-3'	FLAG-TRIM32
CFP-TRIM5α	pECFP-C1	Fw : 5'- ccgctcgagtcgcttctggaatcctggttaatg-3'<br>Rv : 5'- cggaattctcaagagcttggtgagcac-3'	FLAG-TRIM5α
mCherry-TRIM5α	pmCherry-C1	Fw : 5'- ccgctcgagtcgcttctggaatcctggttaatg-3'<br>Rv : 5'- cggaattctcaagagcttggtgagcac-3'	FLAG-TRIM5α
CFP-TRIM63	pECFP-C1	Fw : 5'- gaagatctgattataagtcgagcctgatccag-3'<br>Rv : 5'- acgcgtcgactcactggtgtccttcttccttcc-3'	FLAG-TRIM63
mCherry-TRIM63	pmCherry-C1	Fw : 5'- gaagatctgattataagtcgagcctgatccag-3'<br>Rv : 5'- acgcgtcgactcactggtgtccttcttccttcc-3'	FLAG-TRIM63
CFP-TRIM32(C39S)	pECFP-C1	Fw: 5'-gcttctgcagtctggccatac-3'<br>Rv: 5'-gtatggccagagtgcagaagc-3'	CFP-TRIM32
CFP-TRIM32(K50R)	pECFP-C1	Fw : 5'- cgccagtgcctggagaggctattggccagtagc -3'<br>Rv : 5'-gctactggccaatagcctctccaggcactggcg -3'	CFP-TRIM32
CFP-TRIM32(K401R)	pECFP-C1	Fw : 5'- caagtctttacccgcagaggctttttgaagg -3'<br>Rv : 5'- ccttcaaaaagcctctgcgggtaaagacttg -3'	CFP-TRIM32

CFP-TRIM32(K50R&K401R)	pECFP-C1	Fw : 5'- caagtctttacccgcagaggctttttgaagg -3'<br>Rv : 5'-ccttcaaaaagcctctgcgggtaaagacttg -3'	CFP-TRIM32(K50R)
CFP-TRIM5α(C30S)	pECFP-C1	Fw : 5'- ccctgagcctggactccggccacagcttctg -3'<br>Rv : 5'-cagaagctgtggccggagtccaggctcaggg -3'	CFP-TRIM5α
CFP-TRIM63(C39S)	pECFP-C1	Fw : 5'- ggtcatcttgccgtcccagcacaacctgtg -3'<br>Rv : 5'- cacaggttgtgctgggacggcaagatgacc -3'	CFP-TRIM63

Underlines indicate the sites for restriction enzymes.
