# Supplementary material for: Ubiquitination-coupled liquid phase separation regulates the accumulation of the TRIM family of ubiquitin ligases into cytoplasmic bodies
Source: PLoS One. 2022 Aug 5;17(8):e0272700. doi: 10.1371/journal.pone.0272700 (PMC9355226; doi:10.1371/journal.pone.0272700)
Supplement: S2 Table — (PDF) [file pone.0272700.s002.pdf]

S2 Table List of proteins associated with TRIM32-containg CBs identified LC-MS/MS

Accession number	Protein Mass	Protein description	Ratio[a] (RAF_CB sample /RAF_control sample)
TRI32_HUMAN	71942	E3 ubiquitin-protein ligase TRIM32	261.1
HS90A_HUMAN	84607	Heat shock protein HSP 90-alpha	64.4
HS90B_HUMAN	83212	Heat shock protein HSP 90-beta	62.2
H90B3_HUMAN	68282	Putative heat shock protein HSP 90-beta-3	34.4
LDHB_HUMAN	36615	L-lactate dehydrogenase B chain	33.3
PARP1_HUMAN	113012	Poly [ADP-ribose] polymerase 1	33.3
1433E_HUMAN	29155	14-3-3 protein epsilon	32.0
CH60_HUMAN	61016	60 kDa heat shock protein, mitochondrial	31.1
TBB5_HUMAN	49639	Tubulin beta chain	31.0
VIME_HUMAN	53619	Vimentin	29.0
TBB4B_HUMAN	49799	Tubulin beta-4B chain	25.0
TBA4A_HUMAN	49892	Tubulin alpha-4A chain	24.4
HSP7C_HUMAN	70854	Heat shock cognate 71 kDa protein	23.5
TBB2A_HUMAN	49875	Tubulin beta-2A chain	23.3
TBB2B_HUMAN	49921	Tubulin beta-2B chain	23.3
TBA3C_HUMAN	49928	Tubulin alpha-3C chain	23.0
TBA3D_HUMAN	49928	Tubulin alpha-3D chain	23.0
HS71A_HUMAN	70009	Heat shock 70 kDa protein 1A	22.3
HS71B_HUMAN	70009	Heat shock 70 kDa protein 1B	22.3
RL7_HUMAN	29207	60S ribosomal protein L7	22.2
ADT2_HUMAN	32831	ADP/ATP translocase 2	20.0
ATPA_HUMAN	59714	ATP synthase subunit alpha, mitochondrial	20.0
BIP_HUMAN	72288	Endoplasmic reticulum chaperone BiP	20.0
HNRPC_HUMAN	33650	Heterogeneous nuclear ribonucleoproteins C1/C2	20.0
RL7A_HUMAN	29977	60S ribosomal protein L7a	20.0
RSSA_HUMAN	32833	40S ribosomal protein SA	20.0
LDHA_HUMAN	36665	L-lactate dehydrogenase A chain	19.0
TBA3E_HUMAN	49827	Tubulin alpha-3E chain	18.0
FAS_HUMAN	273254	Fatty acid synthase	17.8
KCRB_HUMAN	42617	Creatine kinase B-type	17.8
PGK1_HUMAN	44586	Phosphoglycerate kinase 1	17.8
RS13_HUMAN	17212	40S ribosomal protein S13	17.8
RS3_HUMAN	26671	40S ribosomal protein S3	17.8
TBB3_HUMAN	50400	Tubulin beta-3 chain	17.8
XRCC5_HUMAN	82652	X-ray repair cross-complementing protein 5	17.8
TBB4A_HUMAN	49554	Tubulin beta-4A chain	17.0
DDX3X_HUMAN	73198	ATP-dependent RNA helicase DDX3X	16.7
IF4A1_HUMAN	46125	Eukaryotic initiation factor 4A-I	16.7
TBA8_HUMAN	50062	Tubulin alpha-8 chain	16.7
TBA1B_HUMAN	50120	Tubulin alpha-1B chain	16.0

TBA1C_HUMAN	49863	Tubulin alpha-1C chain	16.0
TCPB_HUMAN	57452	T-complex protein 1 subunit beta	16.0
H2B2C_HUMAN	21458	Putative histone H2B type 2-C	15.6
H2B2D_HUMAN	18006	Putative histone H2B type 2-D	15.6
HNRPK_HUMAN	50944	Heterogeneous nuclear ribonucleoprotein K	15.6
HNRPU_HUMAN	90528	Heterogeneous nuclear ribonucleoprotein U	15.6
MDHM_HUMAN	35481	Malate dehydrogenase, mitochondrial	15.6
RS4X_HUMAN	29579	40S ribosomal protein S4, X isoform	15.6
STIP1_HUMAN	62599	Stress-induced-phosphoprotein 1	15.6
H4_HUMAN	11360	Histone H4	14.5
TBA1A_HUMAN	50104	Tubulin alpha-1A chain	14.5
ALDOA_HUMAN	39395	Fructose-bisphosphate aldolase A	14.4
ATPB_HUMAN	56525	ATP synthase subunit beta, mitochondrial	14.4
RACK1_HUMAN	35055	Receptor of activated protein C kinase 1	14.4
RL10_HUMAN	24588	60S ribosomal protein L10	14.4
RS2_HUMAN	31305	40S ribosomal protein S2	14.4
TPIS_HUMAN	26653	Triosephosphate isomerase	14.0
A0A2R8Y422_HUMAN	17955	40S ribosomal protein S27a	13.7
RL10L_HUMAN	24503	60S ribosomal protein L10-like	13.3
RLA0_HUMAN	34252	60S acidic ribosomal protein P0	13.3
RS7_HUMAN	22113	40S ribosomal protein S7	13.3
EF2_HUMAN	95277	Elongation factor 2	12.7
ADT3_HUMAN	32845	ADP/ATP translocase 3	12.2
DDX3Y_HUMAN	73108	ATP-dependent RNA helicase DDX3Y	12.2
EF1G_HUMAN	50087	Elongation factor 1-gamma	12.2
H90B2_HUMAN	44321	Putative heat shock protein HSP 90-beta 2	12.2
HS902_HUMAN	39340	Heat shock protein HSP 90-alpha A2	12.2
NPM_HUMAN	32555	Nucleophosmin	12.2
RS16_HUMAN	16435	40S ribosomal protein S16	12.2
SAHH_HUMAN	47685	Adenosylhomocysteinase	12.2
SERA_HUMAN	56614	D-3-phosphoglycerate dehydrogenase	12.2
TCPE_HUMAN	59633	T-complex protein 1 subunit epsilon	12.2
HSP72_HUMAN	69978	Heat shock-related 70 kDa protein 2	12.0
A0A0G2JNQ3_HUMAN	32034	RRM domain-containing protein	11.1
A0A0G2JPF8_HUMAN	32038	RRM domain-containing protein	11.1
ADT1_HUMAN	33043	ADP/ATP translocase 1	11.1
DDX5_HUMAN	69105	Probable ATP-dependent RNA helicase DDX5	11.1
DHX9_HUMAN	140869	ATP-dependent RNA helicase A	11.1
H2AY_HUMAN	39592	Core histone macro-H2A.1	11.1
HNRC1_HUMAN	32123	Heterogeneous nuclear ribonucleoprotein C-like 1	11.1
HNRC2_HUMAN	32053	Heterogeneous nuclear ribonucleoprotein C-like 2	11.1
HNRC3_HUMAN	32010	Heterogeneous nuclear ribonucleoprotein C-like 3	11.1
HNRC4_HUMAN	32010	Heterogeneous nuclear ribonucleoprotein C-like 4	11.1
ILF2_HUMAN	43035	Interleukin enhancer-binding factor 2	11.1

IMA1_HUMAN	57826	Importin subunit alpha-1	11.1
LA_HUMAN	46808	Lupus La protein	11.1
NDKA_HUMAN	17138	Nucleoside diphosphate kinase A	11.1
NDKB_HUMAN	17287	Nucleoside diphosphate kinase B	11.1
PRDX6_HUMAN	25019	Peroxiredoxin-6	11.1
RAN_HUMAN	24408	GTP-binding nuclear protein Ran	11.1
RL15_HUMAN	24131	60S ribosomal protein L15	11.1
RS15A_HUMAN	14830	40S ribosomal protein S15a	11.1
RS18_HUMAN	17708	40S ribosomal protein S18	11.1
SERC_HUMAN	40397	Phosphoserine aminotransferase	11.1
TBA4B_HUMAN	27534	Putative tubulin-like protein alpha-4B	11.1
TCPG_HUMAN	60495	T-complex protein 1 subunit gamma	11.1
TCPQ_HUMAN	59583	T-complex protein 1 subunit theta	11.1
TCPZ_HUMAN	57988	T-complex protein 1 subunit zeta	11.1
H11_HUMAN	21829	Histone H1.1	11.0
H3PS2_HUMAN	15421	Histone HIST2H3PS2	11.0
H31_HUMAN	15394	Histone H3.1	10.7
H12_HUMAN	21352	Histone H1.2	10.5
H3C_HUMAN	15204	Histone H3.3C	10.5
H31T_HUMAN	15499	Histone H3.1t	10.3
H32_HUMAN	15379	Histone H3.2	10.3
H33_HUMAN	15319	Histone H3.3	10.3
RL40_HUMAN	14719	Ubiquitin-60S ribosomal protein L40	10.3
RS27A_HUMAN	17953	Ubiquitin-40S ribosomal protein S27a	10.3
DDX17_HUMAN	80222	Probable ATP-dependent RNA helicase DDX17	10.0
EFTU_HUMAN	49510	Elongation factor Tu, mitochondrial	10.0
H13_HUMAN	22336	Histone H1.3	10.0
H14_HUMAN	21852	Histone H1.4	10.0
HNRH1_HUMAN	49198	Heterogeneous nuclear ribonucleoprotein H	10.0
HNRH2_HUMAN	49232	Heterogeneous nuclear ribonucleoprotein H2	10.0
MCM7_HUMAN	81257	DNA replication licensing factor MCM7	10.0
PTBP1_HUMAN	57186	Polypyrimidine tract-binding protein 1	10.0
RL9_HUMAN	21850	60S ribosomal protein L9	10.0
ROA1_HUMAN	38723	Heterogeneous nuclear ribonucleoprotein A1	10.0
RS14_HUMAN	16263	40S ribosomal protein S14	10.0
RS19_HUMAN	16051	40S ribosomal protein S19	10.0
TCPA_HUMAN	60306	T-complex protein 1 subunit alpha	10.0
TCPH_HUMAN	59329	T-complex protein 1 subunit eta	10.0
UBA1_HUMAN	117774	Ubiquitin-like modifier-activating enzyme 1	10.0
UBB_HUMAN	25746	Polyubiquitin-B	10.0
UBC_HUMAN	76992	Polyubiquitin-C	9.9
HS71L_HUMAN	70331	Heat shock 70 kDa protein 1-like	9.0
RL23A_HUMAN	17684	60S ribosomal protein L23a	9.0
ACLY_HUMAN	120762	ATP-citrate synthase	8.9

DDX21_HUMAN	87290	Nucleolar RNA helicase 2	8.9
GRP75_HUMAN	73635	Stress-70 protein, mitochondrial	8.9
IMB1_HUMAN	97108	Importin subunit beta-1	8.9
PA2G4_HUMAN	43759	Proliferation-associated protein 2G4	8.9
PHB_HUMAN	29786	Prohibitin	8.9
PHB2_HUMAN	33276	Prohibitin-2	8.9
PROF1_HUMAN	15045	Profilin-1	8.9
RL13_HUMAN	24247	60S ribosomal protein L13	8.9
RL18A_HUMAN	20749	60S ribosomal protein L18a	8.9
RL6_HUMAN	32708	60S ribosomal protein L6	8.9
RS4Y1_HUMAN	29437	40S ribosomal protein S4, Y isoform 1	8.9
SFPQ_HUMAN	76102	Splicing factor, proline- and glutamine-rich	8.9
TBB6_HUMAN	49825	Tubulin beta-6 chain	8.9
TCPD_HUMAN	57888	T-complex protein 1 subunit delta	8.9
XRCC6_HUMAN	69799	X-ray repair cross-complementing protein 6	8.9
1433B_HUMAN	28065	14-3-3 protein beta/alpha	8.0
1433T_HUMAN	27747	14-3-3 protein theta	8.0
ACTN1_HUMAN	102993	Alpha-actinin-1	7.8
ACTN4_HUMAN	104788	Alpha-actinin-4	7.8
CLH1_HUMAN	191493	Clathrin heavy chain 1	7.8
DX39A_HUMAN	49098	ATP-dependent RNA helicase DDX39A	7.8
DX39B_HUMAN	48960	Spliceosome RNA helicase DDX39B	7.8
EF1D_HUMAN	31103	Elongation factor 1-delta	7.8
H90B4_HUMAN	58228	Putative heat shock protein HSP 90-beta 4	7.8
HNRPF_HUMAN	45643	Heterogeneous nuclear ribonucleoprotein F	7.8
HS905_HUMAN	38714	Putative heat shock protein HSP 90-alpha A5	7.8
IF4A2_HUMAN	46373	Eukaryotic initiation factor 4A-II	7.8
NDK8_HUMAN	15519	Putative nucleoside diphosphate kinase	7.8
PUR6_HUMAN	47049	Multifunctional protein ADE2	7.8
RL13A_HUMAN	23562	60S ribosomal protein L13a	7.8
RL17_HUMAN	21383	60S ribosomal protein L17	7.8
RL18_HUMAN	21621	60S ribosomal protein L18	7.8
RLA0L_HUMAN	34343	60S acidic ribosomal protein P0-like	7.8
RS20_HUMAN	13364	40S ribosomal protein S20	7.8
RS24_HUMAN	15413	40S ribosomal protein S24	7.8
RS8_HUMAN	24190	40S ribosomal protein S8	7.8
RS9_HUMAN	22578	40S ribosomal protein S9	7.8
TBB8_HUMAN	49744	Tubulin beta-8 chain	7.8
TBB8B_HUMAN	49541	Tubulin beta 8B	7.8
PPIA_HUMAN	18001	Peptidyl-prolyl cis-trans isomerase A	7.3
H2B1B_HUMAN	13942	Histone H2B type 1-B	7.3
H2B1J_HUMAN	13896	Histone H2B type 1-J	7.3
H2B1O_HUMAN	13898	Histone H2B type 1-O	7.3
H2B2E_HUMAN	13912	Histone H2B type 2-E	7.3

H1T_HUMAN	22006	Histone H1t	7.0
H3Y1_HUMAN	15414	Histone H3.Y	7.0
H3Y2_HUMAN	16456	Histone H3.X	7.0
A0A2R8Y4L2_HUMAN	29156	Heterogeneous nuclear ribonucleoprotein A1 pseudogene 48	6.7
ANXA5_HUMAN	35914	Annexin A5	6.7
ASNS_HUMAN	64329	Asparagine synthetase [glutamine-hydrolyzing]	6.7
CDK1_HUMAN	34074	Cyclin-dependent kinase 1	6.7
FUBP2_HUMAN	73070	Far upstream element-binding protein 2	6.7
GDIB_HUMAN	50631	Rab GDP dissociation inhibitor beta	6.7
HNRH3_HUMAN	36903	Heterogeneous nuclear ribonucleoprotein H3	6.7
HNRPM_HUMAN	77464	Heterogeneous nuclear ribonucleoprotein M	6.7
IF2B1_HUMAN	63441	Insulin-like growth factor 2 mRNA-binding protein 1	6.7
IF5A1_HUMAN	16821	Eukaryotic translation initiation factor 5A-1	6.7
MOES_HUMAN	67778	Moesin	6.7
PABP1_HUMAN	70626	Polyadenylate-binding protein 1	6.7
PGAM1_HUMAN	28786	Phosphoglycerate mutase 1	6.7
PLST_HUMAN	70766	Plastin-3	6.7
RA1L2_HUMAN	34204	Heterogeneous nuclear ribonucleoprotein A1-like 2	6.7
RL27_HUMAN	15788	60S ribosomal protein L27	6.7
RL3_HUMAN	46080	60S ribosomal protein L3	6.7
RL4_HUMAN	47667	60S ribosomal protein L4	6.7
H2A1_HUMAN	14083	Histone H2A type 1	6.6
H2A1D_HUMAN	14099	Histone H2A type 1-D	6.6
H2A1H_HUMAN	13898	Histone H2A type 1-H	6.6
H2A1J_HUMAN	13928	Histone H2A type 1-J	6.6
H2AJ_HUMAN	14011	Histone H2A.J	6.6
H2A1B_HUMAN	14127	Histone H2A type 1-B/E	6.5
H2A1C_HUMAN	14097	Histone H2A type 1-C	6.5
H2A2A_HUMAN	14087	Histone H2A type 2-A	6.5
H2A2C_HUMAN	13980	Histone H2A type 2-C	6.5
H2A3_HUMAN	14113	Histone H2A type 3	6.5
1433G_HUMAN	28285	14-3-3 protein gamma	6.0
EF1A1_HUMAN	50109	Elongation factor 1-alpha 1	6.0
EF1A3_HUMAN	50153	Putative elongation factor 1-alpha-like 3	6.0
HSP76_HUMAN	70984	Heat shock 70 kDa protein 6	6.0
SET_HUMAN	33469	Protein SET	6.0
H2AV_HUMAN	13501	Histone H2A.V	5.8
H2AX_HUMAN	15135	Histone H2AX	5.8
H2AZ_HUMAN	13545	Histone H2A.Z	5.8
EF1A2_HUMAN	50438	Elongation factor 1-alpha 2	5.7
H2A1A_HUMAN	14225	Histone H2A type 1-A	5.6
H2B1C_HUMAN	13898	Histone H2B type 1-C/E/F/G/I	5.6
H2B1D_HUMAN	13928	Histone H2B type 1-D	5.6
H2B1H_HUMAN	13884	Histone H2B type 1-H	5.6

H2B1K_HUMAN	13882	Histone H2B type 1-K	5.6
H2B1M_HUMAN	13981	Histone H2B type 1-M	5.6
H2B1N_HUMAN	13914	Histone H2B type 1-N	5.6
H2B2F_HUMAN	13912	Histone H2B type 2-F	5.6
CH10_HUMAN	10925	10 kDa heat shock protein, mitochondrial	5.6
COF1_HUMAN	18491	Cofilin-1	5.6
DEK_HUMAN	42648	Protein DEK	5.6
EZRI_HUMAN	69370	Ezrin	5.6
F10A1_HUMAN	41305	Hsc70-interacting protein	5.6
GBF1_HUMAN	206315	Golgi-specific brefeldin A-resistance guanine nucleotide exchange factor 1	5.6
HS105_HUMAN	96804	Heat shock protein 105 kDa	5.6
IF4A3_HUMAN	46841	Eukaryotic initiation factor 4A-III	5.6
KAD2_HUMAN	26461	Adenylate kinase 2, mitochondrial	5.6
MCM3_HUMAN	90924	DNA replication licensing factor MCM3	5.6
NECT1_HUMAN	---	---	5.6
NONO_HUMAN	54197	Non-POU domain-containing octamer-binding protein	5.6
NUCL_HUMAN	76568	Nucleolin	5.6
PGAM2_HUMAN	28748	Phosphoglycerate mutase 2	5.6
PPIB_HUMAN	23728	Peptidyl-prolyl cis-trans isomerase B	5.6
PSD12_HUMAN	52871	26S proteasome non-ATPase regulatory subunit 12	5.6
PSMD6_HUMAN	45502	26S proteasome non-ATPase regulatory subunit 6	5.6
PUR9_HUMAN	64575	Bifunctional purine biosynthesis protein ATIC	5.6
RADI_HUMAN	68521	Radixin	5.6
RL8_HUMAN	28007	60S ribosomal protein L8	5.6
RS11_HUMAN	18419	40S ribosomal protein S11	5.6
RS25_HUMAN	13734	40S ribosomal protein S25	5.6
RSMB_HUMAN	24594	Small nuclear ribonucleoprotein-associated proteins B and B'	5.6
RSMN_HUMAN	24598	Small nuclear ribonucleoprotein-associated protein N	5.6
ST134_HUMAN	27390	Putative protein FAM10A4	5.6
TBB1_HUMAN	50295	Tubulin beta-1 chain	5.6
TKT_HUMAN	67835	Transketolase	5.6
H2B3B_HUMAN	13900	Histone H2B type 3-B	5.5
1433F_HUMAN	28201	14-3-3 protein eta	5.0
1433S_HUMAN	27757	14-3-3 protein sigma	5.0
RS10_HUMAN	18886	40S ribosomal protein S10	5.0
TPM3_HUMAN	32930	Tropomyosin alpha-3 chain	5.0
TRAP1_HUMAN	80060	Heat shock protein 75 kDa, mitochondrial	5.0
ENOA_HUMAN	47139	Alpha-enolase	4.6
H2B1L_HUMAN	13944	Histone H2B type 1-L	4.6
AATM_HUMAN	47487	Aspartate aminotransferase, mitochondrial	4.4
ACTN2_HUMAN	103788	Alpha-actinin-2	4.4
ADT4_HUMAN	34999	ADP/ATP translocase 4	4.4
C1QBP_HUMAN	31343	Complement component 1 Q subcomponent-binding protein, mitochondrial	4.4
CALX_HUMAN	67526	Calnexin	4.4

DESM_HUMAN	53503	Desmin	4.4
ELAV1_HUMAN	36069	ELAV-like protein 1	4.4
ENPL_HUMAN	92411	Endoplasmin	4.4
F10A5_HUMAN	41351	Putative protein FAM10A5	4.4
FBRL_HUMAN	33763	rRNA 2'-O-methyltransferase fibrillarin	4.4
GLYM_HUMAN	55958	Serine hydroxymethyltransferase, mitochondrial	4.4
H2AW_HUMAN	40033	Core histone macro-H2A.2	4.4
HNRPL_HUMAN	64092	Heterogeneous nuclear ribonucleoprotein L	4.4
IF5A2_HUMAN	16782	Eukaryotic translation initiation factor 5A-2	4.4
IF5AL_HUMAN	16762	Eukaryotic translation initiation factor 5A-1-like	4.4
ILF3_HUMAN	95279	Interleukin enhancer-binding factor 3	4.4
IMDH2_HUMAN	55770	Inosine-5'-monophosphate dehydrogenase 2	4.4
LDH6B_HUMAN	41916	L-lactate dehydrogenase A-like 6B	4.4
LGUL_HUMAN	20764	Lactoylglutathione lyase	4.4
LYAR_HUMAN	43588	Cell growth-regulating nucleolar protein	4.4
MARE1_HUMAN	29980	Microtubule-associated protein RP/EB family member 1	4.4
NACAM_HUMAN	205295	Nascent polypeptide-associated complex subunit alpha, muscle-specific form	4.4
NP1L1_HUMAN	45346	Nucleosome assembly protein 1-like 1	4.4
PABP4_HUMAN	70738	Polyadenylate-binding protein 4	4.4
PGK2_HUMAN	44767	Phosphoglycerate kinase 2	4.4
PSA4_HUMAN	29465	Proteasome subunit alpha type-4	4.4
PSA5_HUMAN	26394	Proteasome subunit alpha type-5	4.4
RAB7A_HUMAN	23475	Ras-related protein Rab-7a	4.4
RBBP4_HUMAN	47626	Histone-binding protein RBBP4	4.4
RL11_HUMAN	20240	60S ribosomal protein L11	4.4
RL12_HUMAN	17808	60S ribosomal protein L12	4.4
RL14_HUMAN	23417	60S ribosomal protein L14	4.4
RL22L_HUMAN	14598	60S ribosomal protein L22-like 1	4.4
RL23_HUMAN	14856	60S ribosomal protein L23	4.4
RL24_HUMAN	17768	60S ribosomal protein L24	4.4
RL26_HUMAN	17248	60S ribosomal protein L26	4.4
RL27A_HUMAN	16551	60S ribosomal protein L27a	4.4
RL29_HUMAN	17741	60S ribosomal protein L29	4.4
RLA2_HUMAN	11658	60S acidic ribosomal protein P2	4.4
ROA2_HUMAN	37407	Heterogeneous nuclear ribonucleoproteins A2/B1	4.4
RS3A_HUMAN	29926	40S ribosomal protein S3a	4.4
SEPT2_HUMAN	41461	Septin-2	4.4
SMD1_HUMAN	13273	Small nuclear ribonucleoprotein Sm D1	4.4
TIF1B_HUMAN	88493	Transcription intermediary factor 1-beta	4.4
UBN1_HUMAN	121446	Ubinuclein-1	4.4
VDAC3_HUMAN	30639	Voltage-dependent anion-selective channel protein 3	4.4
H2BFS_HUMAN	13936	Histone H2B type F-S	4.4
1433Z_HUMAN	27728	14-3-3 protein zeta/delta	4.0
ACTC_HUMAN	41992	Actin, alpha cardiac muscle 1	3.5

ACTS_HUMAN	42024	Actin, alpha skeletal muscle	3.5
H2B1A_HUMAN	14159	Histone H2B type 1-A	3.5
HSP77_HUMAN	40220	Putative heat shock 70 kDa protein 7	3.5
ACTBL_HUMAN	41976	Beta-actin-like protein 2	3.3
2AAA_HUMAN	65267	Serine/threonine-protein phosphatase 2A 65 kDa regulatory subunit A alpha isoform	3.3
AL9A1_HUMAN	53767	4-trimethylaminobutyraldehyde dehydrogenase	3.3
ALDR_HUMAN	35830	Aldo-keto reductase family 1 member B1	3.3
AN32A_HUMAN	28568	Acidic leucine-rich nuclear phosphoprotein 32 family member A	3.3
AN32B_HUMAN	28770	Acidic leucine-rich nuclear phosphoprotein 32 family member B	3.3
ARF4_HUMAN	20498	ADP-ribosylation factor 4	3.3
ATPO_HUMAN	23263	ATP synthase subunit O, mitochondrial	3.3
BYST_HUMAN	49570	Bystin	3.3
C1TC_HUMAN	101495	C-1-tetrahydrofolate synthase, cytoplasmic	3.3
CISY_HUMAN	51680	Citrate synthase, mitochondrial	3.3
CYC_HUMAN	11741	Cytochrome c	3.3
ERF1_HUMAN	49000	Eukaryotic peptide chain release factor subunit 1	3.3
FEN1_HUMAN	42566	Flap endonuclease 1	3.3
GDIA_HUMAN	50550	Rab GDP dissociation inhibitor alpha	3.3
GEMI4_HUMAN	119960	Gem-associated protein 4	3.3
HD_HUMAN	347383	Huntingtin	3.3
HNRPD_HUMAN	38410	Heterogeneous nuclear ribonucleoprotein D0	3.3
HS904_HUMAN	47682	Putative heat shock protein HSP 90-alpha A4	3.3
HSP74_HUMAN	94271	Heat shock 70 kDa protein 4	3.3
IPO5_HUMAN	123550	Importin-5	3.3
MIF_HUMAN	12468	Macrophage migration inhibitory factor	3.3
OAT_HUMAN	48504	Ornithine aminotransferase, mitochondrial	3.3
ODPB_HUMAN	39208	Pyruvate dehydrogenase E1 component subunit beta, mitochondrial	3.3
PAP1L_HUMAN	68349	Polyadenylate-binding protein 1-like	3.3
PDIA6_HUMAN	48091	Protein disulfide-isomerase A6	3.3
PGAM4_HUMAN	28759	Probable phosphoglycerate mutase 4	3.3
PP1A_HUMAN	37488	Serine/threonine-protein phosphatase PP1-alpha catalytic subunit	3.3
PP1B_HUMAN	37163	Serine/threonine-protein phosphatase PP1-beta catalytic subunit	3.3
PP1G_HUMAN	36960	Serine/threonine-protein phosphatase PP1-gamma catalytic subunit	3.3
PSD11_HUMAN	47434	26S proteasome non-ATPase regulatory subunit 11	3.3
PSME3_HUMAN	29488	Proteasome activator complex subunit 3	3.3
PUR2_HUMAN	107699	Trifunctional purine biosynthetic protein adenosine-3	3.3
R13P3_HUMAN	12127	Putative 60S ribosomal protein L13a protein RPL13AP3	3.3
RBBP7_HUMAN	47790	Histone-binding protein RBBP7	3.3
RBMX_HUMAN	42306	RNA-binding motif protein, X chromosome	3.3
RL10A_HUMAN	24816	60S ribosomal protein L10a	3.3
RL22_HUMAN	14778	60S ribosomal protein L22	3.3
RL32_HUMAN	15850	60S ribosomal protein L32	3.3
RL35_HUMAN	14543	60S ribosomal protein L35	3.3
RL5_HUMAN	34341	60S ribosomal protein L5	3.3

ROAA_HUMAN	36202	Heterogeneous nuclear ribonucleoprotein A/B	3.3
RS12_HUMAN	14505	40S ribosomal protein S12	3.3
RS26_HUMAN	13007	40S ribosomal protein S26	3.3
RS4Y2_HUMAN	29277	40S ribosomal protein S4, Y isoform 2	3.3
RU2A_HUMAN	28398	U2 small nuclear ribonucleoprotein A'	3.3
RUVB1_HUMAN	50196	RuvB-like 1	3.3
SEP11_HUMAN	49367	Septin-11	3.3
SEPT6_HUMAN	49685	Septin-6	3.3
SEPT8_HUMAN	55721	Septin-8	3.3
SF3A1_HUMAN	88831	Splicing factor 3A subunit 1	3.3
SMD2_HUMAN	13518	Small nuclear ribonucleoprotein Sm D2	3.3
SP16H_HUMAN	119838	FACT complex subunit SPT16	3.3
SYEP_HUMAN	170483	Bifunctional glutamate/proline--tRNA ligase	3.3
SYLC_HUMAN	134379	Leucine--tRNA ligase, cytoplasmic	3.3
SYTC_HUMAN	83382	Threonine--tRNA ligase 1, cytoplasmic	3.3
SYYC_HUMAN	59106	Tyrosine--tRNA ligase, cytoplasmic	3.3
TERA_HUMAN	89266	Transitional endoplasmic reticulum ATPase	3.3
THOC4_HUMAN	26872	THO complex subunit 4	3.3
TTL12_HUMAN	74356	Tubulin--tyrosine ligase-like protein 12	3.3
UB2L3_HUMAN	17850	Ubiquitin-conjugating enzyme E2 L3	3.3
UB2L5_HUMAN	17864	Ubiquitin-conjugating enzyme E2 L5	3.3
VDAC1_HUMAN	30754	Voltage-dependent anion-selective channel protein 1	3.3
ACTA_HUMAN	41982	Actin, aortic smooth muscle	3.0
ACTH_HUMAN	41850	Actin, gamma-enteric smooth muscle	3.0
H2A2B_HUMAN	13987	Histone H2A type 2-B	3.0
MAST4_HUMAN	283922	Microtubule-associated serine/threonine-protein kinase 4	3.0
PSB1_HUMAN	26472	Proteasome subunit beta type-1	3.0
SETLP_HUMAN	34861	Protein SETSIP	3.0
PRDX4_HUMAN	30521	Peroxiredoxin-4	2.7
ACTBM_HUMAN	41989	Putative beta-actin-like protein 3	2.6
ACTB_HUMAN	41710	Actin, cytoplasmic 1	2.4
ACTG_HUMAN	41766	Actin, cytoplasmic 2	2.4
POTEI_HUMAN	121205	POTE ankyrin domain family member I	2.4
POTEE_HUMAN	121286	POTE ankyrin domain family member E	2.3
POTEF_HUMAN	121367	POTE ankyrin domain family member F	2.3
POTEJ_HUMAN	117315	POTE ankyrin domain family member J	2.3
4F2_HUMAN	67952	4F2 cell-surface antigen heavy chain	2.2
AIFM1_HUMAN	66859	Apoptosis-inducing factor 1, mitochondrial	2.2
ANXA6_HUMAN	75826	Annexin A6	2.2
APT_HUMAN	19595	Adenine phosphoribosyltransferase	2.2
ARF1_HUMAN	20684	ADP-ribosylation factor 1	2.2
ARF3_HUMAN	20588	ADP-ribosylation factor 3	2.2
ARF5_HUMAN	20517	ADP-ribosylation factor 5	2.2
AT1B3_HUMAN	31492	Sodium/potassium-transporting ATPase subunit beta-3	2.2

ATPG_HUMAN	32975	ATP synthase subunit gamma, mitochondrial	2.2
BAF_HUMAN	10052	Barrier-to-autointegration factor	2.2
BCCIP_HUMAN	35957	BRCA2 and CDKN1A-interacting protein	2.2
CAH2_HUMAN	29228	Carbonic anhydrase 2	2.2
CBX3_HUMAN	20798	Chromobox protein homolog 3	2.2
CDK2_HUMAN	33908	Cyclin-dependent kinase 2	2.2
CDK3_HUMAN	35024	Cyclin-dependent kinase 3	2.2
CDN2A_HUMAN	16522	Cyclin-dependent kinase inhibitor 2A	2.2
COF2_HUMAN	18725	Cofilin-2	2.2
COPG1_HUMAN	97655	Coatomer subunit gamma-1	2.2
COPG2_HUMAN	97560	Coatomer subunit gamma-2	2.2
CYBP_HUMAN	26194	Calcyclin-binding protein	2.2
DDTL_HUMAN	14186	D-dopachrome decarboxylase-like protein	2.2
DDX18_HUMAN	75359	ATP-dependent RNA helicase DDX18	2.2
DHX15_HUMAN	90875	Pre-mRNA-splicing factor ATP-dependent RNA helicase DHX15	2.2
DNJA1_HUMAN	44839	DnaJ homolog subfamily A member 1	2.2
DOPD_HUMAN	12704	D-dopachrome decarboxylase	2.2
DRG1_HUMAN	40517	Developmentally-regulated GTP-binding protein 1	2.2
EF1B_HUMAN	24748	Elongation factor 1-beta	2.2
EIF3A_HUMAN	166468	Eukaryotic translation initiation factor 3 subunit A	2.2
EIF3C_HUMAN	105278	Eukaryotic translation initiation factor 3 subunit C	2.2
EIFCL_HUMAN	105407	Eukaryotic translation initiation factor 3 subunit C-like protein	2.2
ERH_HUMAN	12251	Enhancer of rudimentary homolog	2.2
ERP29_HUMAN	28975	Endoplasmic reticulum resident protein 29	2.2
ETFA_HUMAN	35058	Electron transfer flavoprotein subunit alpha, mitochondrial	2.2
F162A_HUMAN	17331	Protein FAM162A	2.2
FKBP4_HUMAN	51772	Peptidyl-prolyl cis-trans isomerase FKBP4	2.2
G6PI_HUMAN	63107	Glucose-6-phosphate isomerase	2.2
GANAB_HUMAN	106807	Neutral alpha-glucosidase AB	2.2
GNA12_HUMAN	44251	Guanine nucleotide-binding protein subunit alpha-12	2.2
GNA13_HUMAN	44022	Guanine nucleotide-binding protein subunit alpha-13	2.2
GSTO1_HUMAN	27548	Glutathione S-transferase omega-1	2.2
HNRDL_HUMAN	46409	Heterogeneous nuclear ribonucleoprotein D-like	2.2
HPRT_HUMAN	24564	Hypoxanthine-guanine phosphoribosyltransferase	2.2
IF5_HUMAN	49192	Eukaryotic translation initiation factor 5	2.2
MATR3_HUMAN	94565	Matrin-3	2.2
MAZ_HUMAN	48576	Myc-associated zinc finger protein	2.2
MCM5_HUMAN	82233	DNA replication licensing factor MCM5	2.2
MPCP_HUMAN	40069	Phosphate carrier protein, mitochondrial	2.2
NACP4_HUMAN	23292	Putative nascent polypeptide-associated complex subunit alpha-like protein	2.2
OLA1_HUMAN	44715	Obg-like ATPase 1	2.2
OST48_HUMAN	50769	Dolichyl-diphosphooligosaccharide--protein glycosyltransferase 48 kDa subunit	2.2
PABP3_HUMAN	69987	Polyadenylate-binding protein 3	2.2
PABP5_HUMAN	43303	Polyadenylate-binding protein 5	2.2

PCBP1_HUMAN	37474	Poly(rC)-binding protein 1	2.2
PDCD5_HUMAN	14276	Programmed cell death protein 5	2.2
PDIA1_HUMAN	57081	Protein disulfide-isomerase	2.2
PERI_HUMAN	53618	Peripherin	2.2
PLSI_HUMAN	70209	Plastin-1	2.2
PLSL_HUMAN	70244	Plastin-2	2.2
PRDX3_HUMAN	27675	Thioredoxin-dependent peroxide reductase, mitochondrial	2.2
PSA1_HUMAN	29537	Proteasome subunit alpha type-1	2.2
PSA2_HUMAN	25882	Proteasome subunit alpha type-2	2.2
PSA3_HUMAN	28415	Proteasome subunit alpha type-3	2.2
PSB5_HUMAN	28462	Proteasome subunit beta type-5	2.2
PSMD3_HUMAN	60939	26S proteasome non-ATPase regulatory subunit 3	2.2
PSMD8_HUMAN	39587	26S proteasome non-ATPase regulatory subunit 8	2.2
RANG_HUMAN	23296	Ran-specific GTPase-activating protein	2.2
RCC1_HUMAN	44941	Regulator of chromosome condensation	2.2
RL21_HUMAN	18553	60S ribosomal protein L21	2.2
RL26L_HUMAN	17246	60S ribosomal protein L26-like 1	2.2
RL28_HUMAN	15738	60S ribosomal protein L28	2.2
RL36_HUMAN	12246	60S ribosomal protein L36	2.2
RL38_HUMAN	8213	60S ribosomal protein L38	2.2
RLA1_HUMAN	11507	60S acidic ribosomal protein P1	2.2
RM12_HUMAN	21335	39S ribosomal protein L12, mitochondrial	2.2
RMXL1_HUMAN	42116	RNA binding motif protein, X-linked-like-1	2.2
RS15_HUMAN	17029	40S ribosomal protein S15	2.2
RS23_HUMAN	15798	40S ribosomal protein S23	2.2
RS26L_HUMAN	12994	Putative 40S ribosomal protein S26-like 1	2.2
RU2B_HUMAN	25470	U2 small nuclear ribonucleoprotein B''	2.2
RUVB2_HUMAN	51125	RuvB-like 2	2.2
SF3B3_HUMAN	135492	Splicing factor 3B subunit 3	2.2
SMD3_HUMAN	13907	Small nuclear ribonucleoprotein Sm D3	2.2
SND1_HUMAN	101934	Staphylococcal nuclease domain-containing protein 1	2.2
SRSF3_HUMAN	19318	Serine/arginine-rich splicing factor 3	2.2
SRSF7_HUMAN	27350	Serine/arginine-rich splicing factor 7	2.2
SRSF9_HUMAN	25526	Serine/arginine-rich splicing factor 9	2.2
SSRP1_HUMAN	81024	FACT complex subunit SSRP1	2.2
SYDC_HUMAN	57100	Aspartate--tRNA ligase, cytoplasmic	2.2
SYVC_HUMAN	140387	Valine--tRNA ligase	2.2
SYWC_HUMAN	53132	Tryptophan--tRNA ligase, cytoplasmic	2.2
TLN1_HUMAN	269599	Talin-1	2.2
TOP2A_HUMAN	174276	DNA topoisomerase 2-alpha	2.2
TRIP6_HUMAN	50255	Thyroid receptor-interacting protein 6	2.2
U5S1_HUMAN	109366	116 kDa U5 small nuclear ribonucleoprotein component	2.2
UBP5_HUMAN	95725	Ubiquitin carboxyl-terminal hydrolase 5	2.2
VATA_HUMAN	68260	V-type proton ATPase catalytic subunit A	2.2

XPO2_HUMAN	110346	Exportin-2	2.2
XPP3_HUMAN	56997	Xaa-Pro aminopeptidase 3	2.2

[a]Ratio of the relative abundance factor (RAF) in CB sample compared with the RAF in control sample (RAF_CB sample/RAF_control sample). The method for calculating RAF values is described under Materials and Methds. Yellow highlights indicate proteins related to Fig. 2C.
