# Supplementary figures and images for: Ubiquitination-coupled liquid phase separation regulates the accumulation of the TRIM family of ubiquitin ligases into cytoplasmic bodies
Source: PLoS One. 2022 Aug 5;17(8):e0272700. doi: 10.1371/journal.pone.0272700 (PMC9355226; doi:10.1371/journal.pone.0272700)

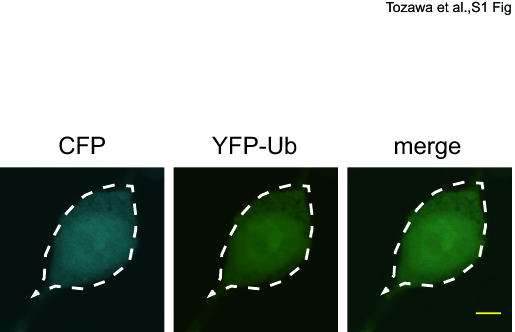

Supplement: S1 Fig — HEK293 cells were co-transfected with CFP and YFP-ubiquitin (YFP-Ub) and visualized by fluorescence microscopy as in Fig 3. Bars, 5 μm. (TIF) [file pone.0272700.s003.tif]

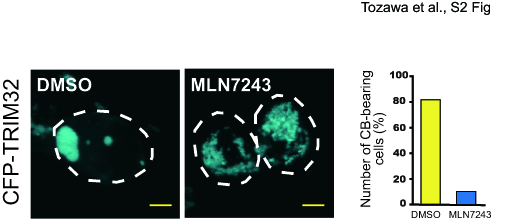

Supplement: S2 Fig — HEK293 cells co-transfected with CFP-TRIM32 and HA-ubiquitin were exposed to DMSO or 5 μM MLN-7242 dissolved in DMSO and analyzed as in Fig 4B. n = 1. Bars, 5 μm. (TIF) [file pone.0272700.s004.tif]
