# Supplementary material for: Ubiquitination-coupled liquid phase separation regulates the accumulation of the TRIM family of ubiquitin ligases into cytoplasmic bodies
Source: PLoS One. 2022 Aug 5;17(8):e0272700. doi: 10.1371/journal.pone.0272700 (PMC9355226; doi:10.1371/journal.pone.0272700)
Supplement: S1 Raw images — (PDF) [file pone.0272700.s005.pdf]

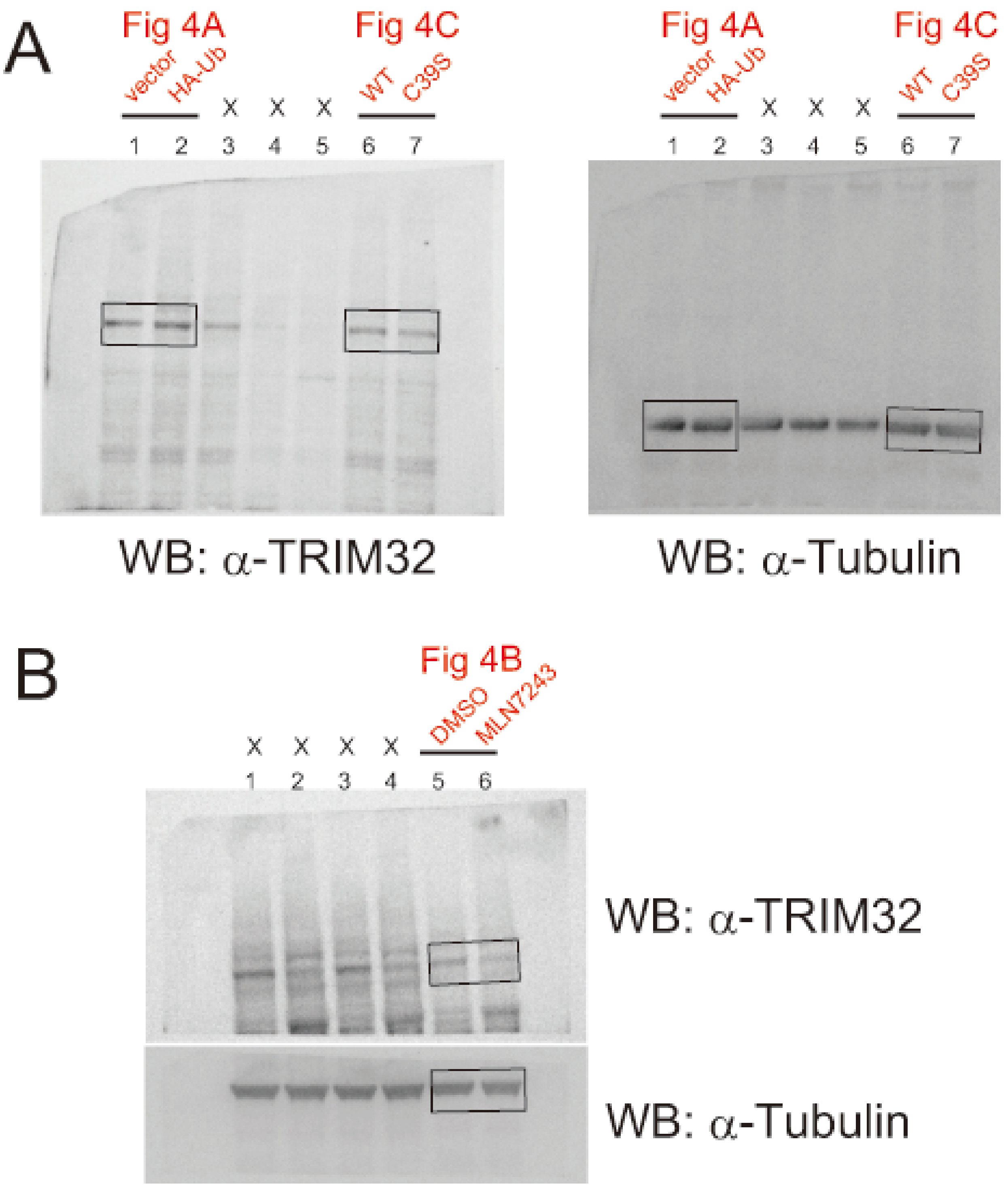

The original blot images of Fig 4. A, The membrane was initially probed with α-TRIM32 and then reprobed with α-tubulin. B. The membrane was cut and probed with α-TRIM32 (upper) and α-tubulin (lower). The protein bands were visualized using secondary antibodies and enhanced chemiluminescence (ECL).
